# Efficacy of natural products on premature ovarian failure: a systematic review and meta-analysis of preclinical studies

**DOI:** 10.1186/s13048-024-01369-5

**Published:** 2024-02-20

**Authors:** Hangqi Hu, Jiacheng Zhang, Xiyan Xin, Yuxin Jin, Yutian Zhu, Haolin Zhang, Ruiwen Fan, Yang Ye, Dong Li

**Affiliations:** Department of Traditional Chinese Medicine, Peking University Third Hospital, 49 North Garden Road, Haidian District, Beijing, 100191 China

**Keywords:** Premature ovarian failure, Phenols, Flavonoids, Oxidative stress, Systematic review

## Abstract

**Objective:**

This study aims to investigate the effects of natural products on animal models of premature ovarian failure (POF).

**Methods:**

We conducted comprehensive literature searches and identified relevant studies that examined the protective effects of natural products on experimental POF. We extracted quantitative data on various aspects such as follicular development, ovarian function, physical indicators, oxidative stress markers, inflammatory factors, and protein changes. The data was analyzed using random-effects meta-analyses, calculating pooled standardized mean differences and 95% confidence intervals. Heterogeneity was assessed using the I^2^ statistic, and bias was estimated using the SYRCLE tool.

**Results:**

Among the 879 reviewed records, 25 articles met our inclusion criteria. These findings demonstrate that treatment with different phytochemicals and marine natural products (flavonoids, phenols, peptides, and alkaloids, etc.) significantly improved various aspects of ovarian function compared to control groups. The treatment led to an increase in follicle count at different stages, elevated levels of key hormones, and a decrease in atretic follicles and hormone levels associated with POF. This therapy also reduced oxidative stress (specifically polyphenols, resveratrol) and apoptotic cell death (particularly flavonoids, chrysin) in ovarian granulosa cells, although it showed no significant impact on inflammatory responses. The certainty of evidence supporting these findings ranged from low to moderate.

**Conclusions:**

Phytochemicals and marine natural product therapy (explicitly flavonoids, phenols, peptides, and alkaloids) has shown potential in enhancing folliculogenesis and improving ovarian function in animal models of POF. These findings provide promising strategies to protect ovarian reserve and reproductive health. Targeting oxidative stress and apoptosis pathways may be the underlying mechanism.

**Supplementary Information:**

The online version contains supplementary material available at 10.1186/s13048-024-01369-5.

## Background

Delayed childbearing worldwide has increased substantially. Unfortunately, women aged 35 and older are more prone to experiencing infertility due to the gradual deterioration of oocyte quality and ovarian reserve [1]. Besides, iatrogenic factors such as surgery, radiotherapy, and chemotherapy also lead to premature follicle depletion [2]. Moreover, the decline in hormone secretions during menopause contributes to various conditions including osteoporosis, Alzheimer’s disease, cardiovascular disease, and increased mortality risk [3]. Given these circumstances, it is crucial to conduct research aimed at developing effective treatments to delay ovarian aging and preserve reproductive health in women.

Both natural and accelerated senescence of the ovaries are associated with peri-menopause syndrome and infertility. This condition, referred to as premature ovarian failure (POF), is characterized by follicle apoptosis, autophagy, atresia, and eventual loss [4, 5]. In POF, abnormal folliculogenesis occurs, leading to the failure of small primordial follicles to mature into preovulatory follicles due to accelerated atresia or aberrant maturation of the follicles [6]. POF affects approximately 1–5% of females under the age of 40, resulting in menstrual disorders, impaired reproductive performance, elevated gonadotropin levels, and decreased estrogen levels [7]. Currently, there is a lack of effective treatments or interventions for POF. Hormone replacement therapy (HRT) is commonly used for clinical management, while oral contraceptive drugs are prescribed for females who do not wish to conceive [8, 9]. In vitro fertilization (IVF) with donor oocytes is an effective approach for addressing infertility in younger females but cannot reverse the impact of aging, particularly in women over 40 years old [10, 11]. Therefore, there is an urgent need for novel and more targeted treatments to address reproductive challenges in women.

Natural products have played a significant role in the development of modern drugs, with their prevalence in top-selling medications increasing since 2006, indicating renewed interest in these structurally diverse compounds for therapeutic purposes [12]. Accumulating evidence from animal models suggests that natural products possess various beneficial properties, including anti-inflammatory, antioxidant, antiproliferative, proapoptotic, and reproductive protective activities [13, 14]. Phytochemicals, in particular, have attracted significant attention due to their potential to scavenge free radicals, exhibit anti-aging effects, regulate cell proliferation, autophagy, and apoptosis pathways, mitigate oxidative stress, and modulate endoplasmic reticulum stress [15–18]. The results of meta-analysises based on clinical evidence suggested that phytotherapy improved pregnancy rates in infertility women compared with placebo treatments [19] and alleviated menstrual disorders, sex hormone levels, and perimenopausal symptoms in primary ovarian insufficiency (POI) patients [20]. A narrative review summarized the effects of natural products (polyphenols, flavonoids, saponins, alkaloids, polysaccharides, etc.) on ovarian function and POF, involving anti-apoptotic, anti-oxidant, anti-aging, immunoregulatory and estrogen-like activities [21]. For instance, isoflavones, often referred to as phytoestrogens, have a potential interaction with estrogen receptors α and β, which may have major adverse impacts on female reproduction [22–24]. And researchers summed up the mechanisms of naturally bioactive ingredients (hyperoside, icaritin, berberine, astragaloside IV, dendrobium nobile polysaccharides, etc.) on inducing proliferation and inhibiting apoptosis of ovarian granulosa cells (GCs), eventually promoting folliculogenesis and steroidogenesis [25]. Besides, it is reported that flavonoids, especially quercetin, improves the quality of oocytes and embryos by affecting the proliferation and apoptosis and decreasing the oxidative stress in GCs [26]. Interestingly, chrysin (5,7-dihydroxyflavone), belonging to flavonoids family, was reported to be effective in preventing POF-induced by chemotherapy and radiotherapy through decreasing oxidative stress and apoptosis [27]. Another natural antioxidant, (-)-epigallocatechin-3-gallate (EGCG) which is a phenolic compound, enhances ovarian function through optimizing ovulation and reducing cyst formation [28]. Moreover, a recent review provides a summary of the primary mechanisms of action through which natural products cause anti-ovarian cancer effects, including cytotoxic effects, damage due to reactive oxygen species (ROS), cell cycle arrest, induction of autophagic cell death, promotion of DNA damage response, etc. [29]. More details of the basis to investigate the efficacy of these natural products on POF can be found in our Table 1.

**Table 1:** Basic information of the included studies

Study (year)	Natural product (type active ingredient study)	Species (age model group (n)/ treatment group (n) weight)	Experimental model method	Natural product intervention (dose duration route)	Outcomes	Potential mechanisms
Mobasher et al. 2023 [30]	Plant, phytochemicals polyphenol, resveratrol	Wistar rats (12–16 weeks, 7/7, 200–240 g)	Intraperitoneal injection daily of Cy for 15 days (200 mg/kg + 8 mg/kg/d)	10 mg/kg/d, 3 weeks, via p.o	(8), (9), (10), (11), (13), (14), (16), (23)	Role of iNOS/Caspase-3 Pathway
Luo et al. 2023 [31]	Marine species marine chemicals peptides, sea cucumber peptide	ICR mouse (8–10 weeks, 3–8/3–8, 30 ± 2 g)	A combined intraperitoneal injection of Cy and BU for 1 day (40 mg/ml + 2.4 mg/ml)	0.6 mg/g/d, 5 weeks, via gavage	(6), (7), (8), (9), (11), (12), (20), (21), (22)	Upregulation the expression of Mapk1, Esr1, Gnrh, StAR, Fshr, and Cyp19a1 due to the activation of the cAMP-related signaling pathways
Moradi et al. 2023 [32]	Plant, phytochemicals alkaloid, capsaicin	Wistar rats (12–16 weeks, 6/6, 210 ± 10 g)	Intraperitoneal injection daily of Cy for 4 days (200 mg/kg + 8 mg/kg/d)	0.5 mg/kg/d, 2 weeks, via i.p	(2), (4), (6), (7), (10), (11), (20), (21)	Upregulation the expression of BAX gene and decrease the expression of apoptosis inducing genes (BCL-2 and P53).
Moradi et al. 2023 [32]	Plant, phytochemicals Flavonol, quercetin	Wistar rats (12–16 weeks, 6/6, 210 ± 10 g)	Intraperitoneal injection daily of Cy for 4 days (200 mg/kg + 8 mg/kg/d)	100 mg/kg/d, 2 weeks, via i.p	(2), (4), (6), (7), (10), (11), (20), (21)	Upregulation the expression of BAX gene and decrease the expression of apoptosis inducing genes (BCL-2 and P53).
İlgen et al. 2023 [33]	Plant, phytochemicals quaternary ammonium salt of an isoquinoline alkaloid, berberine	Wistar rats (not mention, 7/7, 180–220 g)	Intraperitoneal injection daily of Cy for 15 days (50 mg/kg + 8 mg/kg/d)	200 mg/kg/d, 2 weeks, via gavage	(1), (2), (3), (23), (24), (25)	Protective and/or therapeutic effects of reduction proinflammatory cytokines (TNF-α, IL-1, and IL-6)
Xin et al. 2023 [34]	Plant, phytochemicals biphenyls, honokiol	C57BL/6 mice (7 weeks, 5/5, 16–19 g)	A single dose of whole-body gamma radiation (3.2 Gy, XCELL 160) with a dose rate of 0.4975 Gy/min	10 mg/kg/d, 1 week, via i.p	(1), (4), (5), (6), (7), (13), (18), (19), (20), (21), (22), (24)	Inhibition apoptosis and pyroptosis by enhancing HO-1 and Nrf2 expression and nucleus translocation
Wang et al. 2023 [35]	Plant, phytochemicals imidazolidine-2,4-dione, allantoin	Sprague-Dawley rats (not mention, 3–10/3–10, 180–220 g)	Intraperitoneal injection daily of Cy for 21 days (75 mg/kg/d)	140 mg/kg/d, 3 weeks, not mention	(7), (8), (9), (20), (21), (24)	Attenuation of the apoptosis, autophagy, and pyroptosis
Zhang et al. 2022 [36]	Plant, phytochemicals coumarine, daphnetin	C57BL/6 mice (6–8 weeks, 5/5, not mention)	Subcutaneous injection daily of D-gal for 42 days (600 mg/kg/d)	30 mg/kg/d, 6 weeks, via i.p	(1), (2), (3), (4), (5), (7), (8), (14), (16), (18), (19)	Activation of the antioxidant and anti-senescent signaling pathways
Li et al. 2022 [37]	Plant, phytochemicals flavone, chrysin	C57BL/6 mice (7–8 weeks, 20/20, not mention)	Subcutaneous injection daily of D-gal for 42 days (200 mg/kg/d)	144.4 mg/kg/d, not mention, not mention	(2), (3), (4), (6), (7), (8), (10), (11), (13), (14), (17), (20), (21), (22), (23), (24), (25)	Reduction of inflammation and oxidative stress; inhibition of cell apoptosis
Zhao et al. 2022 [38]	Marine species marine chemicals peptides, tilapia skin peptides	C57BL/6 mice (7–8 weeks, 3–15/3–15, not mention)	Intraperitoneal injection daily of Cy for 14 days (10 mg/kg/d)	1000 mg/kg/d, 4 weeks, via gavage	(1), (2), (3), (4), (5), (7), (8), (9), (10), (11), (12), (13), (14), (18), (19), (20), (21), (22)	Inhibition of oxidative stress; regulation Bcl-2/Bax/caspase-3 apoptosis pathway; enhancement of the Nrf2/HO-1 signaling pathway
Wu et al. 2022 [3]	Plant, phytochemicals quaternary ammonium salt of an isoquinoline alkaloid, berberine	Sprague–Dawley rats (not mention, 6/6, 250 g)	Intraperitoneal injection daily of Cy for 15 days (50 mg/kg + 8 mg/kg/d)	400 mg/kg/d, 4 weeks, via gavage	(6), (7), (8), (9), (12)	Inhibition of the PTEN/AKT1/FoxO1 signaling pathway
Barberino et al. 2022 [39]	Plant, phytochemicals flavane, EGCG	Swiss mice (8 weeks, 5/5, 30–35 g)	Intraperitoneal injection singly of Cy for 1 day (200 mg/kg)	50 mg/kg/d, 3 days, via i.p	(1), (2), (5), (16)	Suppression of inflammation and apoptosis; regulation of the Akt/FOXO3a/rpS6 signaling pathway
Zheng et al. 2022 [40]	Plant, phytochemicals Flavonol, quercetin	Sprague-Dawley rats (12 weeks, 15/15, 260 ± 20 g)	Intraperitoneal injection singly of Cy for 1 day (100 mg/kg)	100 mg/kg/d, 6 weeks, via gavage	(1), (2), (3), (4), (5), (6), (7), (8), (9), (12), (13), (14), (15), (17)	Inhibition of oxidative stress level and regulation of the PI3K/Akt/FoxO3a signalling pathway
Ibrahim et al. 2021 [41]	Plant, phytochemicals polyphenol, resveratrol	Wistar rats (12–14 weeks, 8/8, 180–200 g)	Intraperitoneal injection singly of CDDP for 1 day (7 mg/kg)	10 mg/kg/d, 4 weeks, via gavage	(7), (8), (10), (13), (14), (15), (16), (23), (24), (25)	Attenuation of oxidative stress, inflammation and apoptosis
Chen et al. 2021 [42]	Plant, phytochemicals isoflavone, puerarin	Kunming mice (8 weeks, 10/10, 25–30 g)	A combined intraperitoneal injection of Cy and BU for 1 day (120 mg/kg + 30 mg/kg)	200 mg/kg/d, 4 weeks, via gavage	(1), (2), (3), (4), (5), (18), (20), (21)	Involvement of the Wnt/β-catenin signaling pathway, as well as relief of oxidative stress
Jiang et al. 2019 [43]	Plant, phytochemicals polyphenol, resveratrol	Kunming mice (6 weeks, 6/6, not mention)	A combined intraperitoneal injection of Cy and BU for 1 day (120 mg/kg + 30 mg/kg)	40 mg/kg/d, 4 weeks, via gavage	(1), (2), (3), (4), (5), (11), (12), (13), (14), (15), (23), (25)	Relief of oxidative stress and inflammation; involvement of the Hh signaling pathway
Li et al. 2020 [44]	Marine species marine chemicals peptides, oyster polypeptides	C57BL/6 mice (7–8 weeks, 6–10/6–10, 18–22 g)	Subcutaneous injection daily of D-gal for 42 days (200 mg/kg/d)	1000 mg/kg/d, 6 weeks, via gavage	(1), (2), (3), (4), (5), (6), (8), (9), (10), (13), (14), (22)	Rescue of ovarian oxidative damage; reduction of apoptosis
Delkhosh et al. 2019 [45]	Coenzyme in vivo chemicals benzoquinone coenzyme Q10	NMRI mice (8–10 weeks, 8/8, 20–25 g)	Intraperitoneal injection daily of Cy for 21 days (20 mg/kg/d)	22 mg/kg every other day, 3 weeks, via i.p	(1), (2), (4)	Exertion the antioxidant and proliferative properties
Mahran et al. 2019 [46]	Plant, phytochemicals monoterpenoid, carvacrol	Wister rats (23 days, 5–6/5–6, not mention)	A single dose of whole-body gamma radiation (3.2 Gy, LD20) with a doserate of 0.48 Gy/min	80 mg/kg/d, 5 days, via i.p	(6), (7), (8), (12), (23)	Counteraction of oxidative stress and dysregulation of the cross-talk between IGF-1 and TNF-α
Mahran et al. 2019 [46]	Plant, phytochemicals monoterpenoid, thymol	Wister rats (23 days, 5–6/5–6, not mention)	A single dose of whole-body gamma radiation (3.2 Gy, LD20) with a dose rate of 0.48 Gy/min	80 mg/kg/d, 5 days, via i.p	(6), (7), (8), (12), (23)	Counteraction of oxidative stress and dysregulation of the cross-talk between IGF-1 and TNF-α
Li et al. 2019 [47]	Plant, phytochemicals glycosyloxyflavone icariin	C57BL/6 mice (not mention, 10/10, not mention)	Subcutaneous injection daily of D-gal for 42 days (200 mg/kg/d)	100 mg/kg/d, 6 weeks, via i.p	(1), (2), (3), (4), (5), (6), (7), (8), (9)	Promotion of DNA damage repair and attenuation ovarian injury
Mantawy et al. 2019 [27]	Plant, phytochemicals flavone, chrysin	Sprague–Dawley rats (3 weeks, 5–12/5–12, 40–50 g)	A single dose of whole-body gamma-radiation (3.2 Gy, LD20) with a dose rate of 0.48 Gy/min	50 mg/kg/d, 17 days, via p.o	(1), (4), (5), (7), (12), (23)	Downregulation of the TGF-β/MAPK signaling pathway and hinder of inflammatory and apoptotic signal transduction pathways
Yan et al. 2018 [48]	Plant, phytochemicals polyphenol, curcumin	C57BL/6 mice (7–8 weeks, 8–10/8–10, not mention)	Subcutaneous injection daily of D-gal for 42 days (200 mg/kg/d)	100 mg/kg/d, 6 weeks, via i.p	(1), (2), (3), (4), (5), (7), (8), (9), (10), (13), (14), (18), (19), (22)	Inhibition of oxidative stress and apoptosis; regulation of the Nrf2/HO-1 and the PI3K/Akt signaling pathways
Melekoglu et al. 2018 [49]	Plant, phytochemicals polyphenol, curcumin	Wister rats (3–4 months, 10/10, not mention)	Intraperitoneal injection daily of Cy for 15 days (200 mg/kg + 8 mg/kg/d)	100 mg/kg/d, 2 weeks, not mention	(6), (7), (8), (9), (13), (14), (15), (16)	Improvement of oxidative stress marker levels and ovarian reserve marker levels
Melekoglu et al. 2018 [49]	Plant, phytochemicals polyphenol, capsaicin	Wister rats (3–4 months, 10/10, not mention)	Intraperitoneal injection daily of Cy for 15 days (200 mg/kg + 8 mg/kg/d)	0.5 mg/kg/d, 2 weeks, not mention	(6), (7), (8), (9), (13), (14), (15), (16)	Improvement of oxidative stress marker levels and ovarian reserve marker levels
Wu et al. 2018 [50]	Plant, phytochemicals polysaccharides, dendrobium officinal polysaccharides	Kunming mice (15 months, 6–14/6–14, not mention)	NA	70 mg/kg/d, 10 weeks, via p.o	(7), (11), (12), (13), (14), (17), (22), (23), (25)	Reduction of pro-inflammatory cytokines and oxidative stress levels; inhibition of the NF-κB/p53/Bcl-2 mediate signaling pathways; protection and increase of the numbers of mitochondria and endoplasmic reticulum
He et al. 2017 [51]	Plant, phytochemicals Steroid glycosides and triterpene saponins, ginsenoside Rg1	C57BL/6 mice (3 months, 25/25, not mention)	Subcutaneous injection daily of D-gal for 42 days (200 mg/kg/d)	20 mg/kg/d, 4 weeks, via s.c	(1), (3), (4), (6), (7), (8), (11), (13), (14), (17), (23), (24), (25)	Enhancement of the anti-inflammatory and antioxidant capacities; reduction of the expression of senescence signal pathway proteins
Soliman Said et al. 2016 [52]	Plant, phytochemicals polyphenol, resveratrol	Sprague–Dawley rats (23 days, 6–12/6–12, 30–40 g)	A single dose of whole-body gamma-radiation (3.2 Gy, LD20) with a dose rate of 0.48 Gy/min	25 mg/kg/d, 17 days, via p.o	(1), (4), (5), (6), (11), (12)	Inhibition of NF-κB provoked inflammatory cytokines and diminishment of the ovarian inflammation; upregulation of the PPAR-γ and SIRT1 expression

(1): counts of primordial follicles; (2): counts of primary follicles; (3): counts of secondary follicles; (4): counts of antral follicles; (5): counts of atretic follicles; (6): AMH; (7): E2; (8): FHS; (9): LH; (10): P; (11): body weight; (12): ovarian index; (13): MDA; (14): SOD; (15): CAT; (16): GSH; (17): GSH-Px; (18): Nrf2; (19): HO-1; (20): Bax; (21): Bcl-2; (22): GC’s apoptosis; (23): TNF-α; (24): IL-1β; (25): IL-6

*EGCG* Epigallocatechin-3-gallate, *AMH* Anti miillerian hormone, *E2* Estradiol, *FSH* Follicle-stimulating hormone, *LH* Luteinizing hormone, *P* Progesterone, *MDA* Malondialdehyde, *SOD* The activity of superoxide dismutase, *CAT* Catalase, *GSH* Glutathione, *GSH-Px* Glutathione peroxidase, *Nrf-2* Nuclear factor-erythroid 2-related factor 2, *HO-1* Hemeoxy genase-1, *Bax* Bcl-2-associated x, *Bcl-2* B-cell lymphoma-2, *GC* Granulosa cell, *TNF-α* Tumor necrosis factor alpha, *IL-1β* Interleukin 1β, *IL-6* Interleukin 6, *D-gal* D-galactose, *Cy* Cyclophosphamide, *CDDP* Cisplatin (CDDP), *Bu* Busulfan, *GR* Gamma radiation, *Gy* Gray, *NA* Natural aging, *i.p.* Intraperitoneal, *p.o.* Oral, *s.c.* Subcutaneous, *FOXO1* Forkhead box O1, *AKT1* AKT serine/threonine kinase 1, *PTEN* Phosphatase and tensin homologue, *PI3K* Phosphoinositide 3-kinase, *Akt* Protein kinase B; *FoxO3a* Forkhead box O3, *RPS6* Ribosomal protein S6, *Wnt* Wingless/integrated signaling, *β-catenin* Beta-catenin, *Hh* Hedgehog, *IGF-1* Insulin-like growth factor 1, *TGF-β* Transforming growth factor beta, *MAPK* Mitogen-activated protein kinase, *NF-κB* Nuclear factor kappa B, *PPAR-γ* Peroxisome proliferator-activated receptor-gamma, *SIRT1* Sirtuin 1

However, most natural product molecules for POF treatment are still in the early stages of scientific research. In order to facilitate their application on a wider scale, more rigorous clinical trials and in vivo laboratory studies are required. Despite the growing interest, there is currently a lack of systematic reviews that synthesize preclinical evidence on the effects of natural products in treating POF. The aim of this study is to conduct a systematic review and meta-analysis to evaluate the reproductive protective effects and underlying mechanisms of natural products on POF by pooling relevant animal studies.

## Methods

The reporting of this meta-analysis followed the Preferred Reporting Items for Systematic Reviews and Meta-Analysis (PRISMA) guidelines, ensuring transparency and quality of reporting [53]. The study protocol for this research has been registered in the PROSPERO database (CRD42023385100, available at http://www.crd.york.ac.uk/PROSPERO) prior to conducting the study.

### Search strategy

A comprehensive literature search was independently conducted in PubMed, Web of Science, and Scopus databases to identify relevant animal studies published from the inception of these databases up until January 16th, 2024. Only original full-text articles written in English were included in this review. The search strategy utilized a combination of free-text words and Medical Subject Headings (MeSH) terms to capture articles investigating disease models and pharmacological interventions in the titles, abstracts, and keywords. Key search terms used encompassed a wide range of relevant topics, including “Ovarian Diseases”, “Menopause, Premature”, “Ovarian Failure, Premature”, “Premature Ovarian Failure”, “Ovarian Insufficiency, Primary”, “Primary Ovarian Insufficiency”, “Models, Animal”, “Animal Experimentation”, “Animals, Laboratory”, “Natural Products”, “Phytoconstituents”, “Secondary Metabolites”, “Alkaloids”, “Lignins”, “Phenols”, “Coumarins”, “Flavonoids”, “Flavones”, “Flavanols”, “Flavonols”, “Flavanes”, “Isoflavones”, “Saponins”, “Glycosides”, “Terpenoids”, and “Sterols”. The use of a wildcard symbol “*” was employed to broaden the search scope. Additionally, the reference lists of relevant articles were manually screened to ensure the inclusion of all pertinent studies. Detailed descriptions of the electronic search strategies can be found in Table S1.

### Inclusion and exclusion criteria

The review employed the following predefined criteria to include studies: 1) Publication in peer-reviewed academic journals, 2) Availability of full-text articles in English, 3) Use of in vivo animal models of induced preclinical POF, 4) Inclusion of experimental studies with both control design (POF animal models) and treatment design (POF animals treated with natural products), 5) Reporting of at least one of the two primary outcome parameters, specifically hormonal levels and/or follicle counts, 6) Provision of sample size, means, standard deviation, or standard error, and 7) Availability of full-text articles either as open access online or obtainable through author contact.

The following criteria were used to exclude studies from the review: 1) Non-peer-reviewed publications, 2) Non-original research articles, such as letters, reviews, commentaries, editorials, conference proceedings, or method papers that lack primary data, 3) Articles written in languages other than English, 4) Studies conducted on animal species other than mice and rats, 5) Studies unrelated to models of POF, 6) In vitro studies, clinical trials, or in silico research, 7) Interventions other than the administration of a natural compound or mixture, 8) Studies involving concomitant treatments, 9) Studies lacking a control group or where the control group was treated with any drug, and 10) Studies with insufficient outcomes reported or full-text articles that were inaccessible despite author correspondence.

### Data extraction

All references obtained from the literature search were organized and managed using EndNote software. Duplicate records were identified and removed to ensure each study was considered only once. Two review authors independently screened the titles and abstracts of the identified records to determine eligibility for inclusion in the review. If a study appeared to meet the inclusion criteria based on title and abstract, the full-text article was retrieved and thoroughly assessed for inclusion. Any available errata and supplementary materials associated with the included articles were also collected. Any discrepancies or disagreements between the review authors during the selection process were resolved through consensus or consultation with a mentor. The entire process of study selection, including the decisions made at each stage, was documented following the guidelines outlined in the PRISMA statement [54].

The data extraction process was carried out independently by two review authors, utilizing pre-piloted forms in Microsoft Excel. The following key information was carefully extracted from the selected papers: first author, year of publication, animal characteristics (strain, species, age, initial weight), sample size (number of subjects in the treated and control groups), parameters of inducing POF (pharmaceutical agents, modeling methods), intervention characteristics (natural product used, chemical components, doses, route of administration, duration of treatment). The primary outcome measures extracted included hormonal levels (such as anti-Müllerian hormone (AMH), estradiol (E2), follicle-stimulating hormone (FSH), luteinizing hormone (LH), progesterone (P)) and follicle counts (including primordial follicles, primary follicles, secondary follicles, antral follicles, and atretic follicles). The secondary outcome measures extracted included physical characteristics (such as body weight and ovarian index) and potential mechanisms, such as oxidative stress, inflammatory factors, and apoptosis pathways. In cases where a study did not provide the necessary data, the authors were contacted to request the relevant information. Moreover, when multiple doses were administered in a study, data from the highest dose were recorded for the main analysis [55]. If a study evaluated multiple natural products, each treatment was analyzed separately as distinct original research [56]. In cases where the outcomes were presented graphically, Image J software was employed to quantify the results. This rigorous data extraction process ensures accurate and comprehensive collection of relevant information from the included studies.

Response mean values (X_model_ and X_treatment_), standard deviations (S_model_ and S_treatment_), and sample sizes (N_model_ and N_treatment_) were extracted from tables, figures, or text in each included PDF file. When data were presented graphically, image digitization was performed using ImageJ software [57], with each image calibrated to the nearest 0.01 mm. Variance measurements were converted to standard deviations of the mean using MetaWin statistical calculator.

### Risk of bias assessment

The quality assessment of individual publications included in this review was independently performed by two reviewers using the SYRCLE’s Risk of Bias tool. This 10-item scale is specifically designed for evaluating the risk of bias in animal studies [58]. This assessment checklist includes six domains: 1) selection bias (sequence generation, baseline characteristics, and allocation concealment), 2) performance bias (random housing and blinding of trial caregivers), 3) detection bias (random outcome assessment and blinding of outcome assessors), 4) attrition bias (incomplete outcome data), 5) reporting bias (selective out-come reporting), and 6) other bias. Each item in the checklist was assigned a score of 1 point, resulting in a total score of 10 points. Any disagreements that arose during any phase of the project were resolved through negotiation, consensus, or, if required, third-party arbitration.

### Data synthesis and analysis

For data analysis and visualization, we utilized R software (version 4.1.3) along with the “meta” and “dmetar” packages. Since all the outcomes were continuous variables, we employed the standardized mean difference (SMD) with 95% confidence intervals (CI) to standardize the results. The random-effects model was selected to combine the effect sizes. To assess heterogeneity across the literature, we employed Cochran’s Q statistic and the I^2^ statistic [59, 60]. A significance level of *P* < 0.05 was set. When I^2^ > 50%, indicating significant heterogeneity, subgroup analyses were conducted to identify the potential sources of heterogeneity and their effects on the outcome. The potential subgroup criteria considered were the POF induction agents, therapeutic dose, treatment duration, and route of intervention. To ensure the robustness of our results, sensitivity analyses were performed by sequentially excluding individual studies and re-analyzing the data. To evaluate the potential for publication bias, we employed the trim and fill method [61] and Egger’s bias test [62] using the R software.

## Results

### Study selection

**Fig. 1:**
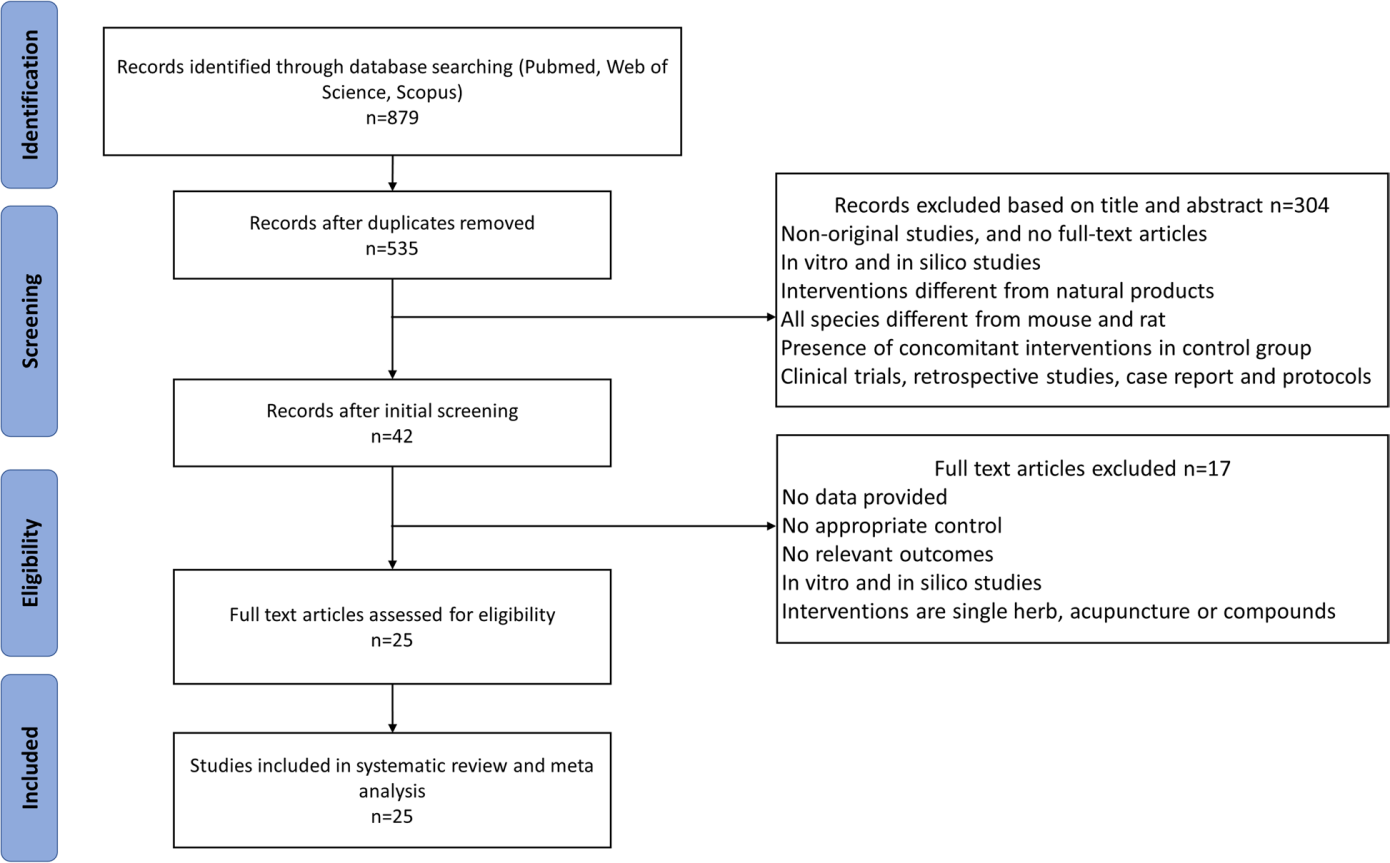
Flow diagram of process of studies inclusion

The detailed process of study selection is presented in Fig. 1. Initially, a search of PubMed, Web of Science, and Scopus databases yielded over 800 records. After excluding non-English publications and removing duplicates, as well as applying the predefined exclusion criteria, a total of 25 eligible studies were identified for inclusion in this meta-analysis examining the effects of natural products on the treatment of POF. This systematic selection process ensures the inclusion of relevant studies meeting the specific criteria outlined in the study protocol.

### Qualitative data

The publication characteristics of the included studies are summarized in Table 1. These eligible studies were published between 2016 and 2023, with the majority conducted by Chinese research teams (*n* = 15). Four papers originated from Egypt, while the remaining studies were from Turkey, Iran, Brazil, and Saudi Arabia, respectively. All studies explicitly stated the use of only female animal subjects. Among the 25 included articles, nine studies utilized mouse models from various inbred and outbred strains, including C57BL/6 (*n* = 8), Kunming (*n* = 3), Swiss (*n* = 1), ICR (*n* = 1) and NMRI (*n* = 1). The remaining studies employed rat strains, specifically Sprague Dawley (*n* = 5) and Wistar (*n* = 6). The sample sizes ranged from 3 to 25 subjects per group.

All comparisons assessing the efficacy of natural products were conducted in animal models of POF. Among these models, 28% utilized aging models, either natural or non-natural, to induce POF. 56% of the studies employed chemotherapy models, using a single drug or combination of drugs, to induce POF. Furthermore, 16% of the studies utilized radiation models to induce POF. The most common methods for inducing POF in the laboratory setting were through the administration of cyclophosphamide (Cy) (40%) and D-galactose (D-gal) (24%). Additionally, 16% of the studies employed gamma radiation (GR) to induce POF, while 12% used a combination of cyclophosphamide and busulfan (Cy and Bu). It is noteworthy that only one study administered a high dose of D-gal (600 mg/kg/day for consecutive 42 days), while the remaining studies used a concentration dose of 200 mg/kg/day administered subcutaneously. Among the studies utilizing Cy, the administration was via intraperitoneal injection alone (40%), with concentrations ranging from 8 to 200 mg/kg, depending on the duration of administration and dosing frequencies. For the radiation-induced POF animal models (16%), a single dose of whole-body gamma radiation was administered at 3.2 Gy (Gy) with a dose rate of 0.48 Gy/min.

In this meta-analysis, twelve studies focused on assessing both follicle counts and hormonal levels as key outcomes of interest. These in vivo parameters were evaluated using techniques such as hematoxylin-eosin staining for follicle counts and enzyme-linked immunosorbent assay (ELISA) for hormonal level measurements. Additionally, several comparisons included evaluations of physical characteristics, such as body weight and ovarian index, to assess the overall impact of natural products on these parameters. Oxidative stress-related targets were assessed in fourteen publications, with most studies measuring the concentration of antioxidant enzymes, such as superoxide dismutase (SOD), or the expression of antioxidant proteins, including nuclear factor-erythroid 2-related factor 2 (Nrf2) and heme oxygenase-1 (HO-1). Other studies examined direct markers of oxidative stress, such as malondialdehyde (MDA), to evaluate the impact of natural products on oxidative stress levels. In addition, several papers characterized inflammation through parameter analyses of key markers, including tumor necrosis factor-alpha (TNF-α), interleukin 1β (IL-1β), and interleukin 6 (IL-6). These evaluations provide insights into the potential anti-inflammatory effects of natural products in the context of POF.

### Treatment agent and dosage

The studies included in this meta-analysis utilized a variety of natural products, representing different classes, components, dosages, and treatment durations. These natural products encompassed substances derived from plants to marine organisms. Among the phytochemicals, bioactive substances from plants were extensively investigated [27, 30, 32–37, 39–43, 46, 47, 49–52, 63]. In addition, marine natural products such as tilapia skin peptides [38], oyster polypeptides [44], sea cucumber peptide [31], and coenzyme Q10 [45] were also explored. Resveratrol was the most commonly selected phytochemicals, administered at doses ranging from 10 to 40 mg/kg. The route of intervention varied significantly among the included studies. Nine studies administered natural products to POF animals via intragastric gavage, four studies used the oral route without further specification, eight studies involved intraperitoneal injection, one study adopted subcutaneous injection, and three studies did not specify the particular route of intervention.

### Quality of included studies

**Fig. 2:**
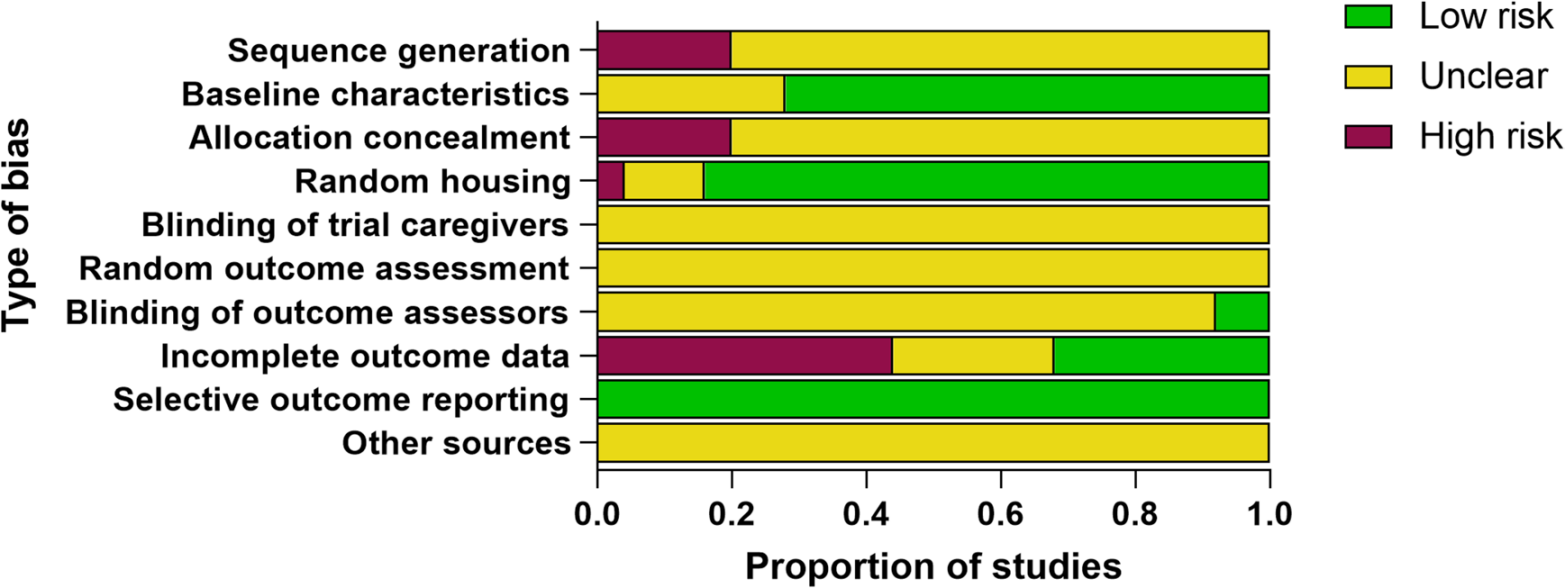
Risk of bias results

The methodological quality of the included studies was assessed using the SYRCLE’s Risk of Bias tool. Overall, the quality of the included literature was found to be relatively low, indicating a potential risk of bias in the majority of the studies. Specifically, while over half of the publications (80%, *n* = 20) described the randomization process for dividing animals into groups, none of the studies provided details on the method and process used to generate the random sequence and allocation concealment. Additionally, 28% of the studies (*n* = 7) did not concretely report animal baseline characteristics, which may introduce potential bias. On the other hand, 84% of the studies (*n* = 21) provided details about random housing, indicating a relatively better adherence to this aspect of study design. Interestingly, all publications (100%, *n* = 25) were free of selective outcome reporting, indicating that the reported outcomes were consistent with the ones originally planned. However, only 8% of the studies (*n* = 2) clarified blinding of outcome assessment, suggesting a potential risk of bias in the assessment of outcome measures. Furthermore, only 32% of the studies (*n* = 8) reported complete outcome data, indicating a potential risk of bias due to incomplete reporting of outcomes. The assessment also revealed a high risk of bias in terms of incomplete outcome reporting. None of the studies provided methodological specifics regarding blinding of experiment caregivers, random outcome assessment, and other potential sources of bias like conflicts of interest. Consequently, the risk of bias for these aspects was deemed unclear (unclear risk of bias = 100%) (Fig. 2).

## Synthesis of results (meta-analysis)

### Primary outcomes

#### Follicular development

**Fig. 3:**
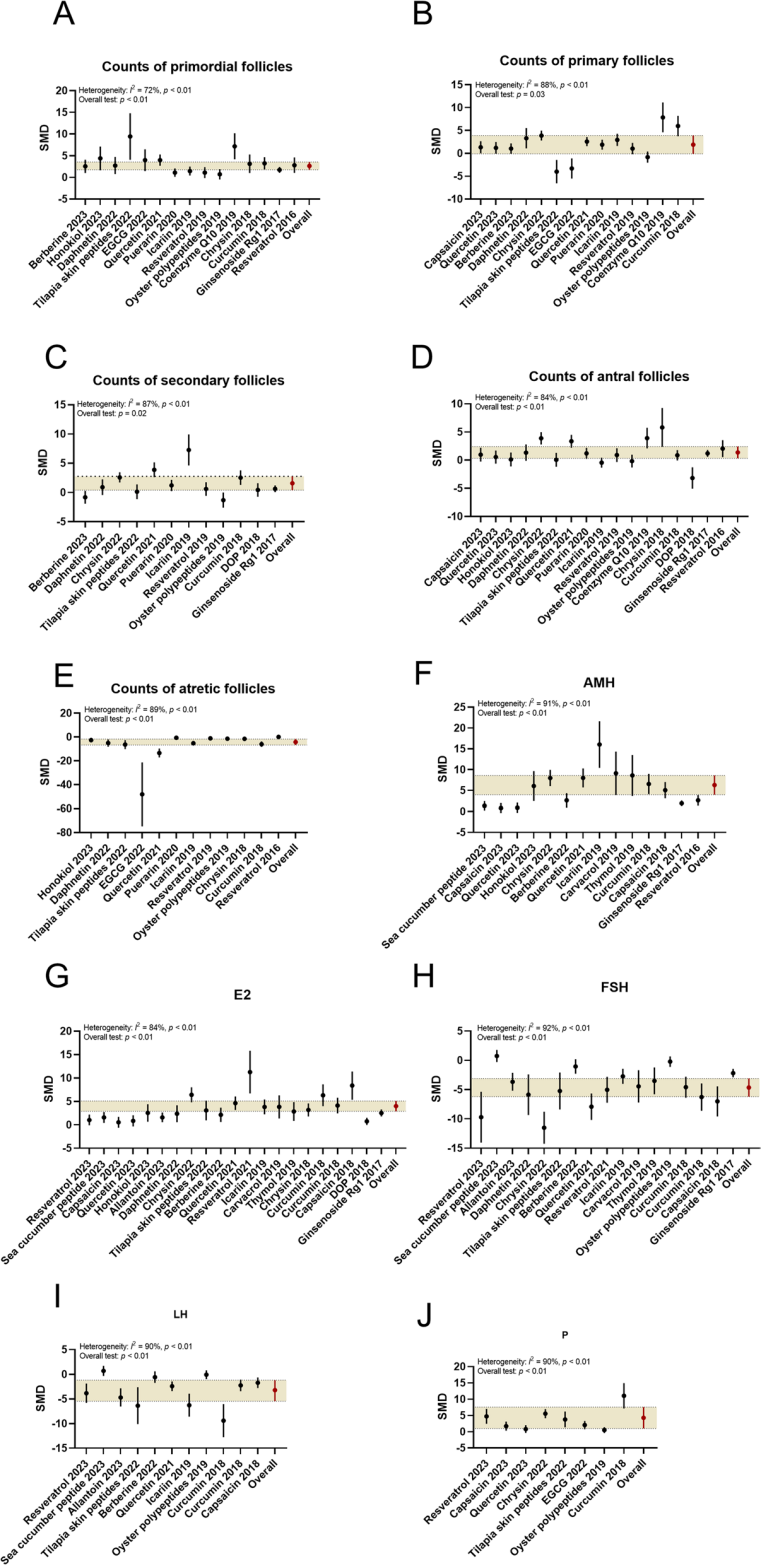
Forest plots: summarizing effects of various natural products on the primary outcomes. **A** Counts of primordial follicles, **B** Counts of primary follicles, **C** Counts of secondary follicles, **D** Counts of antral follicles, **E** Counts of atretic follicles, **F** AMH, **G** E2, **H** FSH, **I** LH, **J** P. The vertical error bars represent the 95% CI for the individual estimates, and the horizontal yellow bars represent the 95% CI of the pooled estimate of efficacy. SMD indicates standardized mean difference

The meta-analysis findings revealed significant protective effects of natural products (flavonoids, peptides, polyphenols, alkaloids, etc.) on ovarian follicular development in the POF model. Treatment with natural products (quercetin, tilapia skin peptides, icariin, resveratrol, etc.) was associated with increased numbers of primordial follicles (SMD: 2.68, 95% CI: 1.89, 3.48, *P* < 0.01; heterogeneity: I^2^ = 72%, *P* < 0.01; Fig. 3), primary follicles (SMD: 1.72, 95% CI: 0.21, 3.24, *P* < 0.05; heterogeneity: I^2^ = 88%, *P* < 0.01), secondary follicles (SMD: 1.37, 95% CI: 0.23, 2.51, *P* < 0.05; heterogeneity: I^2^ = 87%, *P* < 0.01), and antral follicles (SMD: 1.20, 95% CI: 0.36, 2.03, *P* < 0.01; heterogeneity: I^2^ = 84%, *P* < 0.01) in the treatment groups. Additionally, the number of atretic follicles was significantly reduced in the treatment groups compared to the POF model groups (SMD: − 4.01, 95% CI: − 6.24, − 1.77, *P* < 0.01; heterogeneity: I^2^ = 89%, *P* < 0.01; Fig. 3).

#### Ovarian function

The meta-analysis revealed significant protective effects of natural products (flavonoids, polyphenols, alkaloids, etc.) on ovarian function, as indicated by the pooled mean estimates presented in Fig. 3. Compared to the POF model groups, treatment with natural products (icariin, chrysin, resveratrol, curcumin, etc.) significantly increased the levels of AMH (SMD: 4.87 (95% CI: 2.76, 6.97), *P* < 0.01; heterogeneity: I^2^ = 91%, *P* < 0.01), E2 (SMD: 3.16 (95% CI: 2.23, 4.08), *P* < 0.01; heterogeneity: I^2^ = 84%, *P* < 0.01), and P (SMD: 3.44 (95% CI: 1.36; 5.53), *P* < 0.01; heterogeneity: I^2^ = 90%, *P* < 0.01) in the treatment groups. Additionally, the levels of FSH (SMD: − 4.48 (95% CI: − 6.01, − 2.95), *P* < 0.01; heterogeneity: I^2^ = 92%, *P* < 0.01) and LH (SMD: − 3.07 (95% CI: − 4.77, − 1.37), *P* < 0.01; heterogeneity: I^2^ = 90%, *P* < 0.01) were significantly reduced in the treatment groups compared to the POF groups.

### Secondary outcomes

#### Physical characteristics

**Fig. 4:**
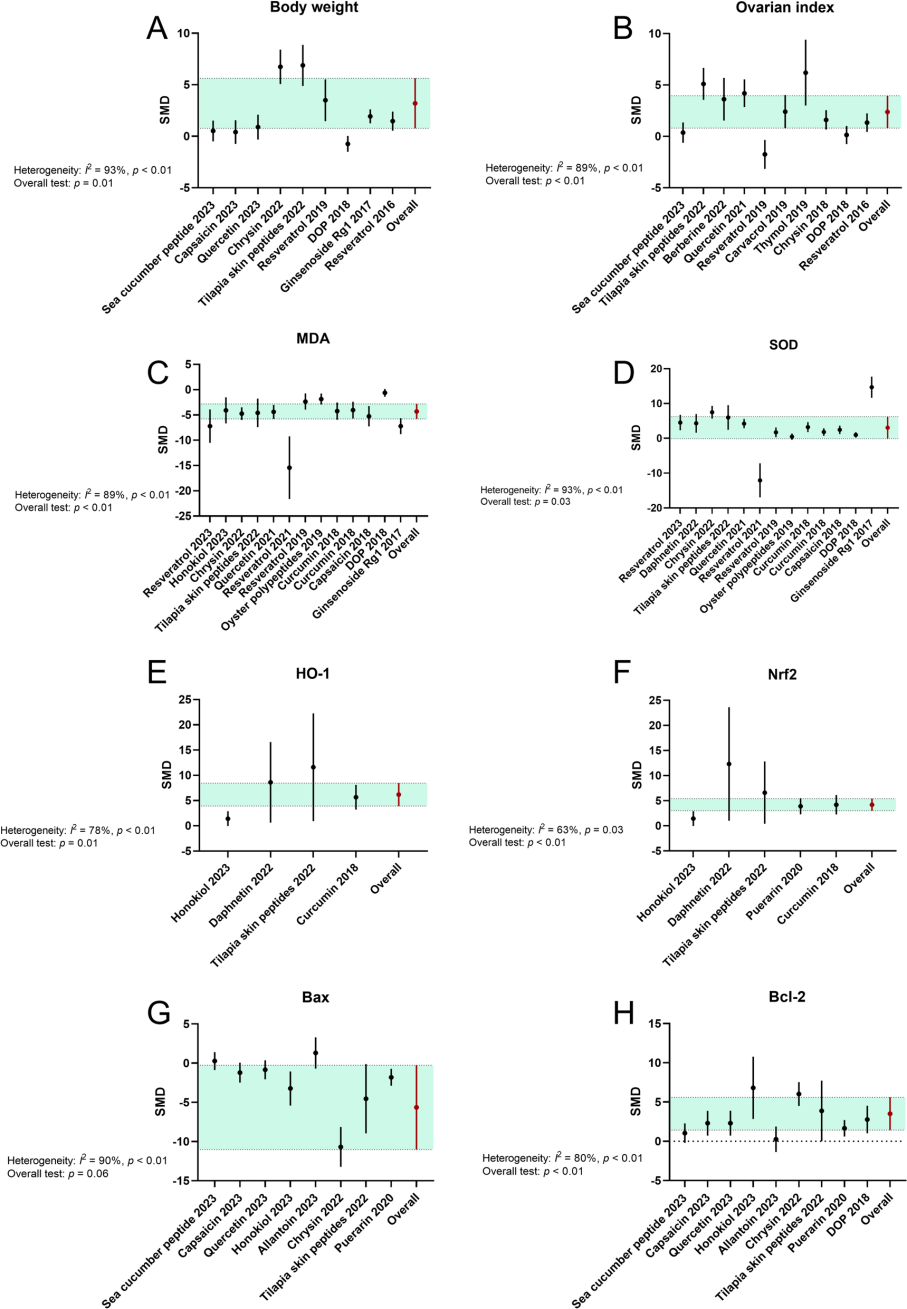
Forest plots: summarizing effects of various natural products on the secondary outcomes. **A** Body weight, **B** Ovarian index, **C** MDA, **D** SOD, **E** HO-1, **F** Nrf2, **G** Bax, **H** Bcl-2. The vertical error bars represent the 95% CI for the individual estimates, and the horizontal green bars represent the 95% CI of the pooled estimate of efficacy. SMD indicates standardized mean difference

More than one-third of the included studies (flavonoids, peptides, monoterpenoid, etc.) compared the treatment effects on physical parameters among animal subjects, and separate analyses were conducted accordingly. The pooled effect sizes demonstrated that supplementation with natural products (chrysin, tilapia skin peptides, thymol, etc.) significantly increased the body weight (SMD: 2.30 (95% CI: 0.55, 4.06), *P* = 0.01; heterogeneity: I^2^ = 93%, *P* < 0.01; Fig. 4) and ovarian index (SMD: 2.16 (95% CI: 0.71, 3.61), *P* < 0.01; heterogeneity: I^2^ = 89%, *P* < 0.01) of animals in the treatment groups compared to those in the POF groups.

#### Oxidative stress

ROS can induce apoptosis of GCs and/or oocytes, leading to follicular atresia and a decline in ovarian reserve function [64]. Our meta-analyses confirmed significant reductions in oxidative stress-related targets associated with the administration of natural products (polyphenols, steroid glycosides and triterpene saponins, ect.) (Fig. 4). Out of the 25 analyzed studies, 12 demonstrated a statistically significant reduction in the MDA level (SMD: − 4.44 (95% CI: − 5.76, − 3.12), *P* < 0.01; heterogeneity: I^2^ = 89%, *P* < 0.01) in the treatment groups (resveratrol, ginsenoside Rg1, ect.) when compared to the models. The SOD level was significantly higher in POF animals that had been treated with natural products (resveratrol, ginsenoside Rg1, ect.) across 13 papers (SMD: 3.17 (95% CI: 0.27, 6.07), *P* = 0.03; heterogeneity: I^2^ = 93%, *P* < 0.01). However, there was no significant evidence regarding the efficacy of natural products in altering the levels of catalase (CAT) and glutathione (GSH) in POF animals (Supplementary materials). Interestingly, the effect on the glutathione peroxidase (GSH-Px) level was found to be significantly favorable in the treatment groups (Fig. S1.1-S1.3).

#### Nrf2/HO-1 pathway

As a crucial cytoprotective transcription factor, Nrf2 plays a significant role in the removal of ROS and subsequent attenuation of inflammatory responses. Nrf2 can initiate the transcription of several cytoprotective and antioxidant genes, including HO-1 [65, 66]. The potential of natural products (coumarine, peptides, etc.) to activate the Nrf2/HO-1 pathway was investigated in at least three studies where the protein expression levels were evaluated as endpoints. The meta-analysis demonstrated a significant increase in Nrf2 protein expression (SMD = 3.57 (95% CI: 1.78, 5.35), *P* < 0.01; heterogeneity: I^2^ = 63%, *P* = 0.03; Fig. 4) and HO-1 protein expression (SMD = 5.09 (95% CI: 1.12, 9.06), *P* = 0.01; heterogeneity: I^2^ = 78%, *P* < 0.01) upon treatment with natural products (daphnetin, tilapia skin peptides, etc.).

#### Apoptosis pathway

The atresia of follicles is a histological manifestation of ongoing cell death, and apoptosis is a cell death mechanism involved in the regulation of ovarian reserve [67, 68]. Various assays, such as the evaluation of apoptosis-relevant proteins or the TUNEL assay, are commonly employed to detect cell death. Among the nine comparisons, treatment with natural products (honokiol, chrysin, etc.) significantly enhanced the expression of B-cell lym-phoma-2 (Bcl-2) protein (SMD = 2.71 (95% CI: 1.39, 4.03), *P* < 0.01; heterogeneity: I^2^ = 80%, *P* < 0.01; Fig. 4). Conversely, the expression of Bcl-2-associated x (Bax) protein was reduced in POF animals medicated with natural products (SMD =  − 2.44 (95% CI: − 4.99, 0.10), *P* = 0.06; heterogeneity: I^2^ = 90%, *P* < 0.01; Fig. 4). Additionally, in four other studies utilizing the TUNEL assay, a significant decrease in apoptotic granulosa cells in antral follicles of POF patterns was observed following supplementation with natural products (such as curcumin) (Fig. S1.4).

#### Pro-inflammatory biomarker

The depletion of the follicular reserve in POF may be influenced by various genetic, environmental, or therapeutic factors that can activate extracellular signal-regulated kinases and trigger the release of pro-inflammatory cytokines such as TNF-α, IL-1β, and IL-6. However, this meta-analysis did not detect significant differences in the levels of TNF-α, IL-1β, and IL-6 between the model and treatment groups (Fig. S1.5-S1.7).

### Sensitivity analysis

A sensitivity analysis was conducted for the primary outcomes related to ovarian follicular development and ovarian function. Systematic removal of articles one-by-one did not explain the heterogeneity or lead to significant deviations in the results related to follicle count, except for primary follicles. It is important to note that the difference in the count of primary follicles was no longer significant with the removal of three studies, specifically “Zhang et al., 2022”, “Li et al., 2022”, and “Yan et al., 2018”. In terms of hormone levels, sequential elimination of each article from the meta-analysis did not yield substantial discrepancies between the pre- and post-sensitivity analysis pooled estimates (Fig. S2.1-S2.2).

### Subgroup analyses

**Fig. 5:**
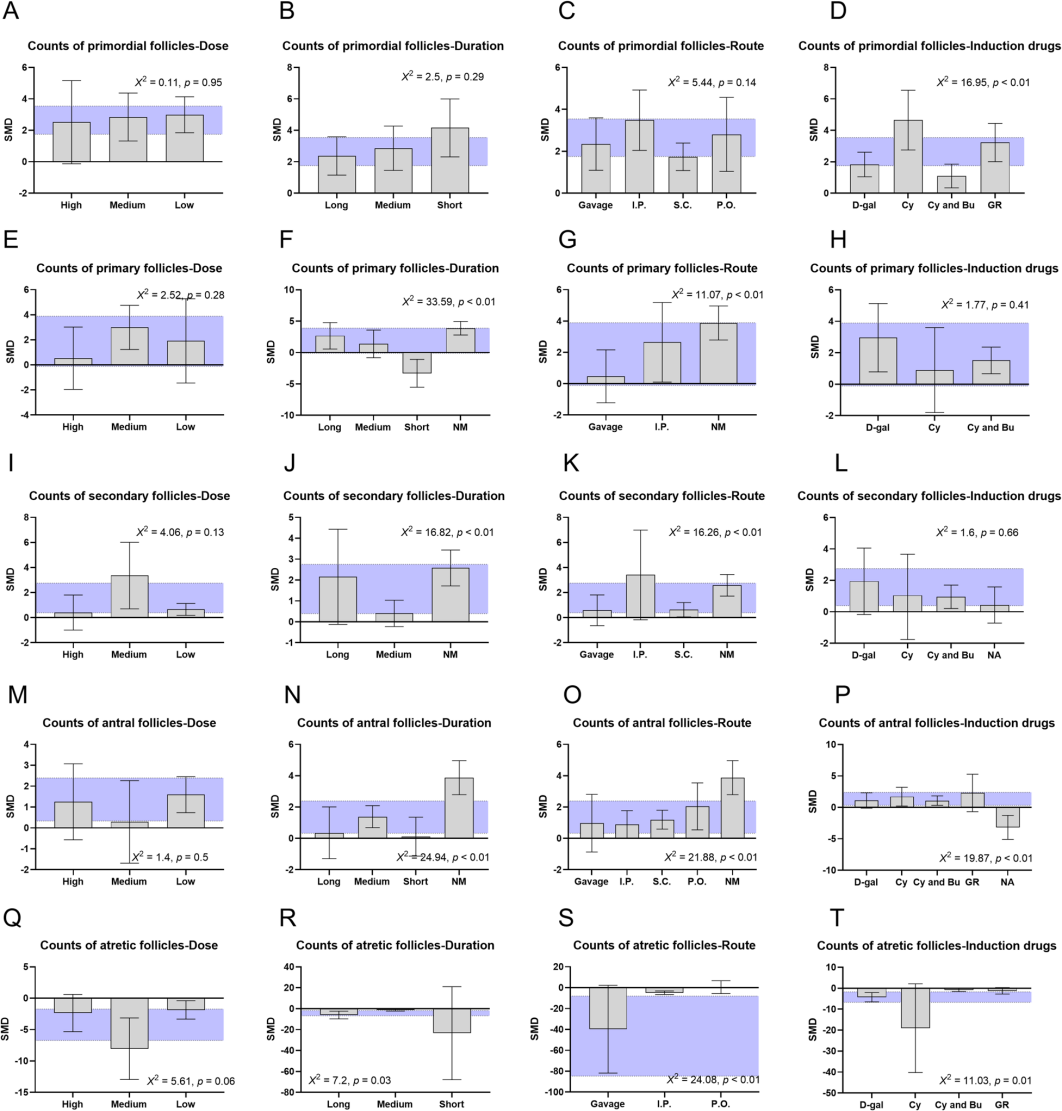
Bar graph: subgroup analysis of therapeutic dose, treatment duration, route of intervention, and induction agents. **A** to **D** Counts of primordial follicles, **E** to **H** Counts of primary follicles, **I** to **L** Counts of secondary follicles, **M** to **P** Counts of antral follicles, **Q** to **T** Counts of atretic follicles. The vertical error bars represent the 95% CI for the subgroup estimates, and the horizontal purple bars represent the 95% CI of the pooled estimate of efficacy. SMD indicates standardized mean difference

Most of the outcome measures demonstrated at least moderate heterogeneity (I^2^ > 50%). To investigate the potential sources of variability among the included studies, a series of subgroup analyses were conducted (Fig. 5). The counts of primordial follicles, primary follicles, secondary follicles, antral follicles, and atretic follicles, as well as the levels of various hormones, were the primary variables of interest, given their relevance to the effects of natural products on POF. Several predefined categorical variables were considered in the subgroup analyses to explore the variations in experimental methods. These variables included the therapeutic dose of the natural products, treatment duration, route of intervention, and the specific agents used for inducing POF.

For primordial follicles, the source of heterogeneity among the articles was related to the “induction agents”. The effect size was statistically better in subjects that POF animal models were induced by the Cy (*p* < 0.01; Fig. 5). Regarding primary follicles, both “treatment duration” and “route of intervention” were identified as sources of heterogeneity among the papers (*p* < 0.01, *p* < 0.01). Notably, the effect size of natural products on the count of primary follicles was worse in those POF animals medicated with a relatively short treatment duration. In the case of secondary follicles, significant correlations were also observed between the “treatment duration” and “route of intervention”, and the effect size (*p* < 0.01, *p* < 0.01). For antral follicles, a short treatment duration and natural aging POF model did have a significant worse impact on the effect size (*p* < 0.01, *p* < 0.01). Concerning atretic follicles, studies with a long treatment duration, gavage administration route, and those utilizing a Cy-induced POF model showed statistically superior effect size (*p* = 0.03, *p* < 0.01, *p* = 0.01, respectively; Fig. 5).

**Fig. 6:**
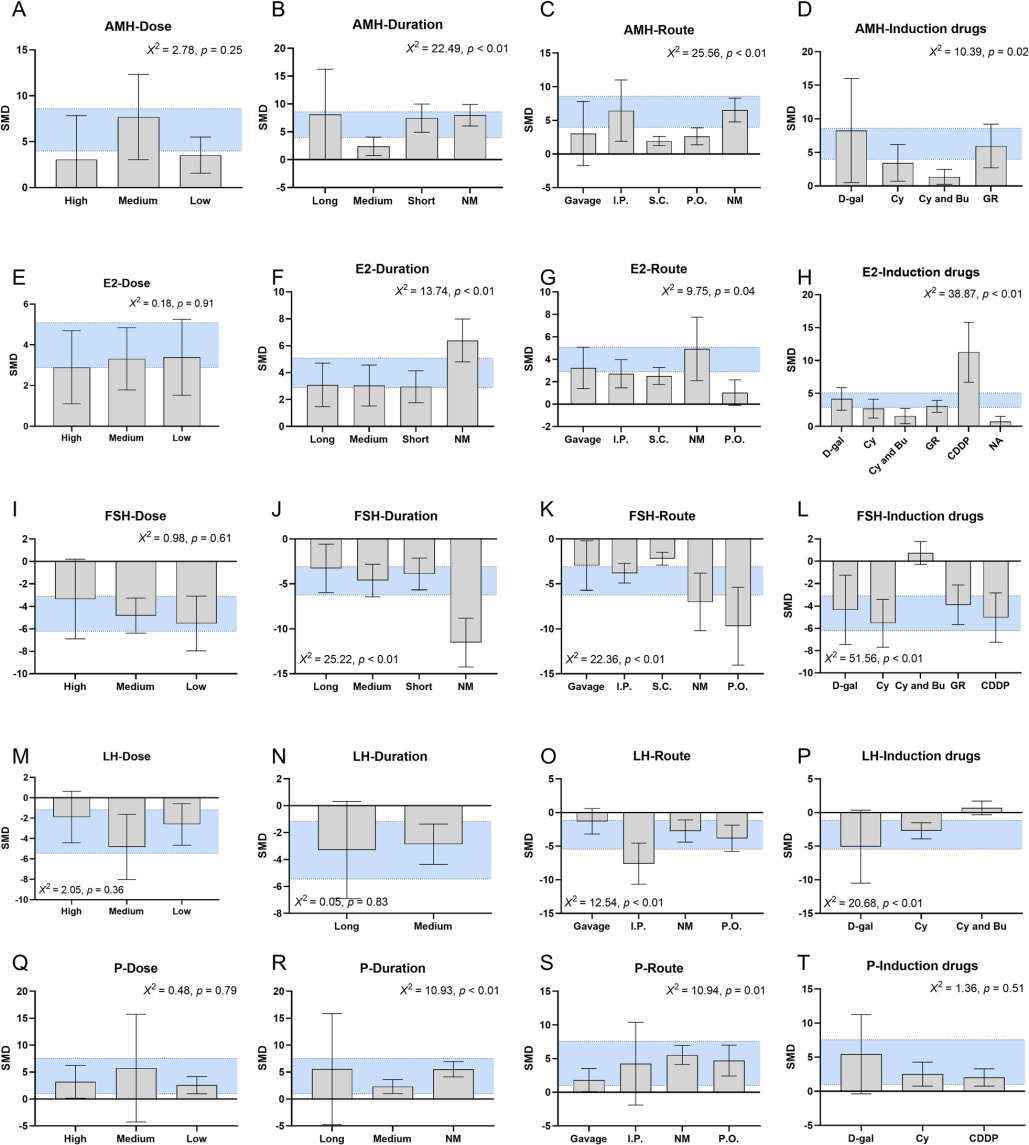
Bar graph: subgroup analysis of therapeutic dose, treatment duration, route of intervention, and induction agents. **A** to **D** AMH level, **E** to **H** E2 level, **I** to **L** FSH level, **M** to **P** LH level, **Q** to **T** P level. The vertical error bars represent the 95% CI for the subgroup estimates, and the horizontal blue bars represent the 95% CI of the pooled estimate of efficacy. SMD indicates standardized mean difference

The subgroup analysis demonstrated significant effects on the AMH level, particularly in the subgroup categorized as “medium” treatment duration, “s.c.” route of intervention, “Cy and Bu” induction agents (*p* < 0.01, *p* < 0.01, *p* = 0.02, respectively; Fig. 6). As for the E2 level, the “treatment duration”, “route of intervention”, and “induction agents” were identified as sources of heterogeneity among the literature (*p* < 0.01, *p* = 0.04, *p* < 0.01, respectively). Similarly, for the FSH level, “treatment duration”, “route of intervention”, and “induction agents” also were recognized as sources of heterogeneity among the papers (*p* < 0.01, *p* < 0.01, *p* < 0.01, respectively). For the LH level, significant effects were demonstrated in studies utilizing the “i.p.” route of intervention and “D-gal” induction agents, compared to those using “gavage” and “Cy and Bu” (*p* < 0.01, *p* < 0.01). For the P level, it showed heterogeneity among the publications, with the “treatment duration” and “route of intervention” being the identified sources (*p* < 0.01, *p* = 0.01; Fig. 6). Additionally, subgroup analyses were conducted to determine whether the animal strain was a source of heterogeneity in the primary outcomes. However, these analyses revealed scarcely any significant statistical differences among the publications that successfully established POF models using different rodent strains (Fig. S2.3).

## Publication bias

**Fig. 7:**
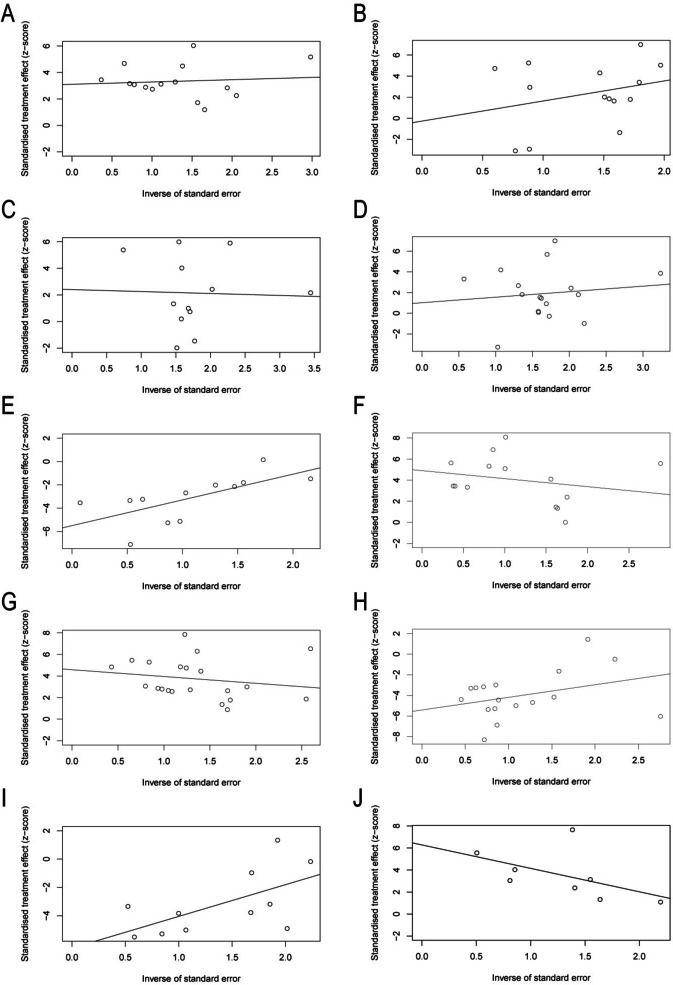
Egger’s publication bias for **A** counts of primordial follicles, **B** counts of primary follicles, **C** counts of secondary follicles, **D** counts of antral follicles, **E** counts of atretic follicles, **F** AMH, **G** E2, **H** FSH, **I** LH, **J** P

**Fig. 8:**
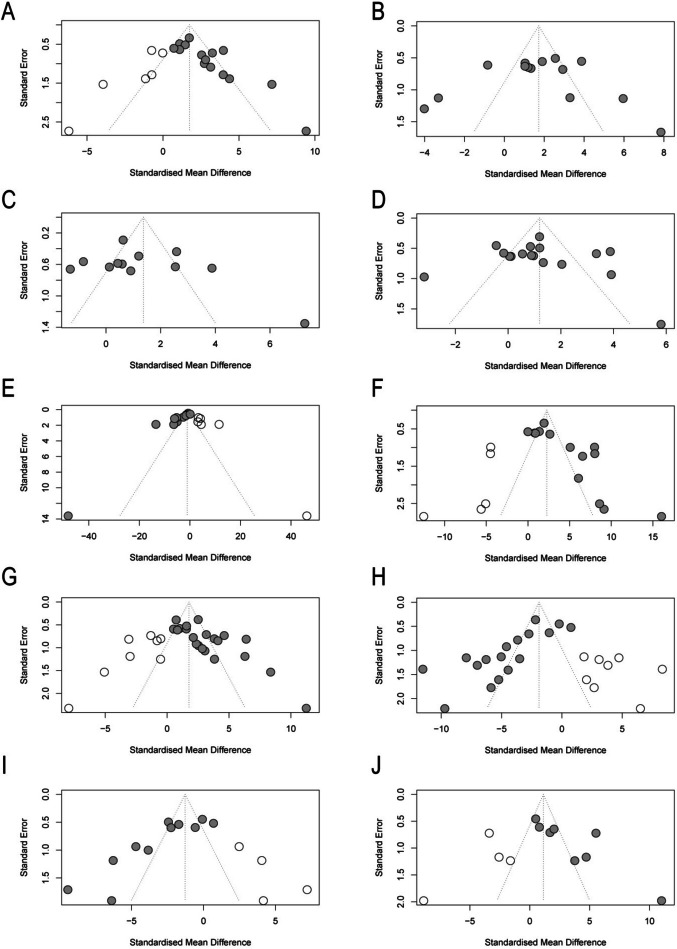
Trim-and-fill analysis for **A** counts of primordial follicles, **B** counts of primary follicles, **C** counts of secondary follicles, **D** counts of antral follicles, **E** counts of atretic follicles, **F** AMH, **G** E2, **H** FSH, **I** LH, **J** P

Publication bias was assessed through a random-effects model, funnel plots, Egger regression for asymmetry, and trim-and-fill analysis. Publication bias analysis is most convincing with far greater than 5 literatures, which were available for most of the primary outcomes. Although the number of included studies was sufficient for most of the primary outcomes, funnel plot asymmetry was detected using Egger regression for follicular development (primordial follicle, *P* = 0.0017; primary follicle, *P* = 0.92; secondary follicle, *P* = 0.363; antral follicle, *P* = 0.608; and atretic follicle, *P* = 0.0002) and ovarian function (AMH, *P* = 0.0016; and E2, *P* = 0.0005; FSH, *P* = 0.0003; LH, *P* = 0.0023; P, *P* = 0.0183) (Fig. 7). Trim-and-fill analysis revealed the addition of researches or no change in open arm/center time (Fig. 8).

## Discussion

In this systematic review and meta-analysis, we examined the potential therapeutic effects of naturally bioactive substances on animal models of experimental POF-induced infertility. Our findings indicate that the administration of natural products leads to several positive outcomes, including increased ovarian weight and ovarian index, restored ovarian follicle numbers, elevated levels of AMH, E2, and P, as well as decreased levels of FSH and LH. These results strongly suggest that the administration of natural products has the potential to effectively ameliorate the condition of animals with POF. The beneficial effects of these natural products are likely attributed to their antioxidant defense mechanisms and their ability to suppress apoptosis. These bioactive substances can help protect the ovarian reserve and enhance the fertility potential of the female reproductive system. Furthermore, through subgroup analyses, we explored potential sources of heterogeneity in the primary outcomes. The variation in therapeutic dose, treatment duration, route of intervention, and induction agents among the included studies may contribute to the observed heterogeneity. These factors should be taken into consideration when interpreting the results and designing future studies in this field.

The choice of induction agent has a direct impact on the reduction of atretic follicles and the increase in primordial follicles. Most studies included in this review used chemotherapy alkylating triggers [69] or excessive glucose stimulation [70, 71] to establish POF models, which provide reliable and reproducible experimental conditions that mimic the pathogenesis of POF, including immune and senescence mechanisms [72]. However, it is important to note that the ovarian damage caused by cyclophosphamide is self-reversible within a certain time period [73, 74]. Furthermore, the route of intervention significantly affects serum hormone concentrations, and differences in dissolution and metabolism can contribute to observed disparities [75, 76]. The protective effects of natural products also vary depending on the therapeutic doses administered. However, inconsistent measurement and reporting of treatment duration and route of intervention make it difficult to replicate studies and understand the true impact of bioactive substances on POF. It is worth noting that the classified and structurally diverse agents derived from natural products exhibit varying therapeutic and preventive activities [75]. Unfortunately, limited subgroup analysis is available in the current literature, hampering definitive conclusions based on existing evidence. Recent research highlights that the effects of natural products depend significantly on their (micro)environment, cell type, and nanoformulation, allowing for the development of various drug delivery systems [77]. Previous systematic reviews and meta-analyses have identified factors such as the induction agent, type of natural product, route of drug delivery, animal model, treatment dosage, and duration of administration as potential sources of substantial heterogeneity between studies [78, 79].

Although the therapeutic effects of natural products on animal models of POF have been demonstrated, the precise mechanisms underlying the restoration of ovarian reserve are not fully understood. Current evidence suggests that ovarian vascularization lesions, apoptosis, ROS, and accelerated inflammation are underlying mechanisms involved in POF-related ovarian dysfunction [80–82]. Impaired ovarian reserve, the primary defect caused by chemotherapeutic agents, is characterized by excessive apoptosis of granulosa cells (GCs) and follicle depletion, leading to ovarian failure [83]. Two biological pathways, the mitochondrial pathway and the death receptor pathway, play essential roles in GC apoptosis [84]. Our findings confirm that the administration of bioactive compounds leads to increased levels of the pro-apoptotic marker Bax and decreased levels of the anti-apoptotic marker Bcl-2 in the ovarian tissue of POF animals. Bax and Bcl-2, as members of the Bcl-2 family, are crucial regulators of the mitochondrial apoptosis pathway in the ovaries [85]. Bax, mainly located in the cytoplasm, promotes apoptosis by translocating to the mitochondrial membrane, releasing cytochrome C (cyt-C), and accelerating follicular atresia. Conversely, Bcl-2 forms heterodimers with Bax and exerts an anti-apoptotic effect [86–88]. These findings suggest that the administration of natural products may regulate the balance between pro-apoptotic and anti-apoptotic markers, potentially inhibiting excessive apoptosis and promoting the survival of ovarian follicles.

Increased oxidative stress in ovarian tissues is a key factor in promoting apoptosis in granulosa cells (GCs) [89, 90]. ROS, which play important roles in cellular growth and metabolism, accumulate within cells when the body’s antioxidant defense system is overwhelmed. These intracellular ROS attack organelles and biomolecules, resulting in varying degrees of oxidative stress damage to DNA, lipids, and proteins, thereby triggering GC death through various pathways [91]. In this context, the intracellular ovarian environment is disrupted, impeding nuclear and cytoplasmic maturation of oocytes and triggering apoptosis, thereby disturbing the balance between ROS production and removal. Consequently, the accumulation of ROS in the ovaries impairs oocyte quality and causes rapid degeneration of the corpus luteum [92]. Furthermore, the amplification of lipid peroxidation cascades in the ovaries results in oxidative damage, significantly impacting folliculogenesis, oocyte meiosis, ovulation, and ultimately contributing to ovarian failure [93].

The reproductive system is particularly vulnerable to oxidative damage. In the ovaries, the production of free radicals increases while the levels of antioxidants de-crease, significantly impairing the ability to scavenge free radicals [70]. Nrf2, a critical factor for maintaining redox equilibrium, regulates the expression of downstream antioxidant enzymes such as HO-1, thereby protecting cells from injury caused by excessive ROS [94]. Three to four studies included in our meta-analysis examined the expressions of Nrf2 and HO-1 in ovarian tissues of POF animals, suggesting the protective effects of natural compounds against oxidative stress. Consistent with current reports, MDA is the end product of oxygen radical-induced lipid peroxidation, reflecting oxidative damage in mammals under conditions of antioxidant deficiency. Conversely, key antioxidant enzymes or molecules such as SOD, GSH-Px, or CAT can protect the ovaries from oxidative stress damage [95, 96]. Therefore, more than ten literatures measured the peripheral levels of these parameters, demonstrating some improvements in the treated subjects. These findings collectively suggest that bioactive products have beneficial effects specifically on oxidative stress markers, restoring the balance between oxidative outcomes and antioxidant defenses in POF.

Regarding other parameters such as histopathology, follicle counts are commonly considered crucial determinants of ovarian capacity and generally reflect ovarian lifespan. Overactivation or depletion of ovarian follicles can lead to POF. In terms of serum hormone concentrations, most papers in our systematic review reported correlations with female fertility outcomes, evaluated using various methods. AMH is widely regarded as the preferred quantitative marker for ovarian reserve function and predicting POF [97, 98]. Additionally, serum levels of FSH, LH, and E2 are essential for the diagnosis of POI, and they are typically influenced by the menstrual cycle in clinical practice [99]. Notably, over ten studies identified improvements in these factors, mainly assessed through follicle counts and serum hormone measurements. Overall, considering all the outcomes from our systematic review and meta-analysis, natural products demonstrate a counteracting effect on experimentally induced ovarian damage, making a significant contribution to female fertility.

Contemporarily, HRT and cryopreservation techniques combined with assisted reproductive technology (ART) treatments are commonly applied to improve oocyte quality and preserve fertility in women with POF [100]. However, it is important to note that each technique for treating POF has its inherent drawbacks. Hormone therapies, despite being effective in managing symptoms and preventing complications, carry a high risk of cancer or recurrence in organs such as the ovaries and endometrium [100]. They are also associated with an increased risk of stroke and cardiovascular disease. On the other hand, fertility preservation techniques, although offering the possibility of preserving fertility, are often expensive and have lower success rates, making them an incomplete solution for all patients [101]. In light of these limitations, the development of effective therapies for POF is an ongoing area of research. Natural molecular medicines, derived from various plants, animals, or microbes, have been instrumental in maintaining global health and serve as the basis for many commercial medications [102]. Compared to early commercial drugs, natural biomedicines offer several advantages, including better safety, stability, efficacy, lower side effects, and reduced risk of resistance. In this systematic review and meta-analysis, flavonoids, a diverse group of natural phytochemicals known for their biological and pharmacological properties, emerged as the most abundant group of polyphenolic compounds used in the treatment of POF [103]. Flavonoids have been found to reduce ROS production, restore mitochondrial function, and promote anti-apoptotic activity, thereby aiding in the preservation of ovarian function. Among these flavonoids, quercetin has demonstrated potent antioxidant properties. Its antioxidant capacity is attributed to its ability to directly scavenge ROS, chelate metal ions, and modulate various antioxidant defense systems. Studies have shown that pre-supplementation with quercetin can protect nuclear morphology, restore the balance between pro-apoptotic and anti-apoptotic markers, inhibit caspase signaling activation, and reduce the expression of p53 [104]. Furthermore, quercetin has been shown to protect against Cyclophosphamide-induced ovarian damage by reversing mitochondrial dysfunction and promoting mitochondrial biogenesis through the proliferator-activated receptor γ coactivator-1α (PGC-1α) pathway. Additionally, quercetin supplementation may improve ovarian function by downregulating pyroptosis, a form of programmed cell death [105]. Therefore, quercetin is commonly considered as a potential agent for treating POF. Interestingly, most of the literature indicates that the beneficial effects of natural products are dose-dependent, with higher doses generally being more effective than lower doses. However, subgroup analyses have shown that therapeutic dose is not the source of heterogeneity, and protective effects on ovarian reserve retention and oxidative stress mitigation even at medium to low doses of natural products. This suggests that even at lower doses, natural products can have a positive impact on POF.

Nevertheless, it is important to acknowledge the limitations of our research. Firstly, the eligible studies included in our research exhibit variations in methodology, including the use of different animal models, induction agents, and treatment details. This heterogeneity makes it challenging to thoroughly analyze the influence of each methodological parameter. Secondly, the studies are grouped into three categories based on therapeutic dose or treatment duration in our review. However, the wide range of intake observed within these groups may hinder definitive conclusions regarding the optimal dosage or duration of administration. Thirdly, the heterogeneity in both methods and outcomes among the included studies is a challenging factor in the progress of infertility therapies. It makes it difficult to draw consistent conclusions and limits the generalizability of the findings. Lastly, this study did not include papers published in languages other than English, which may lead to potential publication bias in our research.

## Conclusion

In summary, pooling evidence from 25 preclinical articles, we have observed the protective effects of phytochemicals and marine natural compounds (explicitly phenols, flavonoids, alkaloids, and peptides) in POF, improving folliculogenesis and ovarian function through mechanisms such as inhibition of oxidative stress and apoptosis, and increased cytoprotective and antioxidant markers. The effect of supplementation is influenced by treatment duration, intervention route, therapeutic doses, and induction agents used. This meta-analysis is the first compilation of evidence on natural products as therapeutic agents for POF, providing a valuable foundation for further research and potentially impacting women reproductive health and the development of effective POF therapies.

### Supplementary Information

**Additional file 1: Supplemental Table 1.** Search strategies for PubMed, Web of Science, and Scopus. **Supplemental Figure 1.1.** Forest plots: effects of various natural products on the secondary outcome of CAT level. **Supplemental Figure 1.2.** Forest plots: effects of various natural products on the secondary outcome of GSH level. **Supplemental Figure 1.3.** Forest plots: effects of various natural products on the secondary outcome of GSH-Px level. **Supplemental Figure 1.4.** Forest plots: effects of various natural products on the secondary outcome of GC’s apoptosis. **Supplemental Figure 1.5.** Forest plots: effects of various natural products on the secondary outcome of TNF-a level. **Supplemental Figure 1.6.** Forest plots: effects of various natural products on the secondary outcome of IL-β level. **Supplemental Figure 1.7.** Forest plots: effects of various natural products on the secondary outcome of IL-6 level. **Supplemental Figure 2.1.** Sensitivity analysis of various natural products on the primary outcomes of follicular development. (A) counts of primordial follicles, (B) counts of primary follicles, (C) counts of secondary follicles, (D) counts of antral follicles, (E) counts of atretic follicles. **Supplemental Figure 2.2.** Sensitivity analysis of various natural products on the primary outcomes of ovarian function. (A) AMH, (B) E2, (C) FSH, (D) LH, (E) P. **Supplemental Figure 2.3.** Forest plots: subgroup analysis of the strains. (A) counts of primordial follicles, (B) counts of primary follicles, (C) counts of secondary follicles, (D) counts of antral follicles, (E) counts of atretic follicles, (F) AMH, (G) E2, (H) FSH, (I) LH, (J) P.

## Data Availability

No datasets were generated or analysed during the current study.

## Abbreviations

AMH: Anti miillerian hormone
Akt: Protein kinase B
AKT1: AKT serine/threonine kinase 1
ART: Assisted reproductive technology
Bax: Bcl-2-associated x
Bcl-2: B-cell lymphoma-2
Bu: Busulfan
CAT: Catalase
CDDP: Cisplatin
CI: Confidence intervals
Cy: Cyclophosphamide
Cyt-C: Cytochrome C
D-gal: D-galactose
E2: Estradiol
EGCG: Epigallocatechin-3-gallate
FOXO1: Forkhead box O1
FoxO3a: Forkhead box O3
FSH: Follicle-stimulating hormone
GC: Granulosa cell
GR: Gamma radiation
GSH: Glutathione
GSH-Px: Glutathione peroxidase
HO-1: Hemeoxy genase-1
HRT: Hormone replacement therapy
IGF-1: Insulin-like growth factor 1
IL-1β: Interleukin 1β
IL-6: Interleukin 6
IVF: In vitro fertilization
LH: Luteinizing hormone
MAPK: Mitogen-activated protein kinase
MDA: Malondialdehyde
NA: Natural aging
NF-κB: Nuclear factor kappa B
Nrf-2: Nuclear factor-erythroid 2-related factor 2
P: Progesterone
PGC-1α: Peroxisome proliferator‑activated receptor γ coactivator‑1α
PI3K: Phosphoinositide 3-kinase
POF: Premature ovarian failure
POI: Primary ovarian insufficiency
PPAR-γ: Peroxisome proliferator-activated receptor-gamma
PTEN: Phosphatase and tensin homologue
ROS: Reactive oxygen species
RPS6: Ribosomal protein S6
SIRT1: Sirtuin 1
SMD: Standardized mean differences
SOD: Superoxide dismutase
TGF-β: Transforming growth factor beta
TNF-α: Tumor necrosis factor alpha
Wnt: Wingless/integrated signaling
