# Supplementary material for: Efficacy of natural products on premature ovarian failure: a systematic review and meta-analysis of preclinical studies
Source: J Ovarian Res. 2024 Feb 20;17:46. doi: 10.1186/s13048-024-01369-5 (PMC10877904; doi:10.1186/s13048-024-01369-5)
Supplement: Supplementary file 1 — Additional file 1: Supplemental Table 1. Search strategies for PubMed, Web of Science, and Scopus. Supplemental Figure 1.1. Forest plots: effects of various natural products on the secondary outcome of CAT level. Supplemental Figure 1.2. Forest plots: effects of various natural products on the secondary outcome of GSH level. Supplemental Figure 1.3. Forest plots: effects of various natural products on the secondary outcome of GSH-Px level. Supplemental Figure 1.4. Forest plots: effects of various natural products on the secondary outcome of GC’s apoptosis. Supplemental Figure 1.5. Forest plots: effects of various natural products on the secondary outcome of TNF-a level. Supplemental Figure 1.6. Forest plots: effects of various natural products on the secondary outcome of IL-β level. Supplemental Figure 1.7. Forest plots: effects of various natural products on the secondary outcome of IL-6 level. Supplemental Figure 2.1. Sensitivity analysis of various natural products on the primary outcomes of follicular development. (A) counts of primordial follicles, (B) counts of primary follicles, (C) counts of secondary follicles, (D) counts of antral follicles, (E) counts of atretic follicles. Supplemental Figure 2.2. Sensitivity analysis of various natural products on the primary outcomes of ovarian function. (A) AMH, (B) E2, (C) FSH, (D) LH, (E) P. Supplemental Figure 2.3. Forest plots: subgroup analysis of the strains. (A) counts of primordial follicles, (B) counts of primary follicles, (C) counts of secondary follicles, (D) counts of antral follicles, (E) counts of atretic follicles, (F) AMH, (G) E2, (H) FSH, (I) LH, (J) P. [file 13048_2024_1369_MOESM1_ESM.pdf]

Supplementary Materials

1. Supplemental Table

Table S1. Search strategies for PubMed, Web of Science, and Scopus.

Number	Search terms
1	"Animals" [Mesh] OR "Models, Animal" [Mesh] OR "Animal Experimentation" [Mesh] OR "Animals, Laboratory" [Mesh]
2	"Animal" [Title/Abstract] OR "Animals" [Title/Abstract] OR "Animal Model" [Title/Abstract] OR "Animal Models" [Title/Abstract] OR "preclinical studies" [Title/Abstract] OR "rat" [Title/Abstract] OR "rats" [Title/Abstract] OR "mouse" [Title/Abstract] OR "mice" [Title/Abstract] OR "rodent" [Title/Abstract]
3	1 OR 2
4	"Ovarian Failure, Premature" [MeSH] OR "Ovarian Insufficiency, Primary" [MeSH] OR "Menopause, Premature" [MeSH] OR "Ovarian Diseases" [MeSH] OR "Fertility" [MeSH] OR "Ovarian Follicle" [MeSH] OR "Ovarian reserve"
5	"Premature ovarian failure" [Title/Abstract] OR "premature ovary failure" [Title/Abstract] OR "POF" [Title/Abstract] OR "Primary ovarian insufficiency" [Title/Abstract] OR "primary ovary insufficiency" [Title/Abstract] OR "POI" [Title/Abstract] OR "fertility" [Title/Abstract] OR "fertile" [Title/Abstract] OR "fertilities" [Title/Abstract] OR "ovarian" [Title/Abstract] OR "ovarians" [Title/Abstract] OR "ovar* and (failure or function or damage)" [Title/Abstract]
6	4 OR 6

7	"Biological Products" [MeSH] OR "Plant Preparations"
8	"natural product" [Title/Abstract] OR "natural products" [Title/Abstract] OR "plant natural products" [Title/Abstract] OR "marine natural products" [Title/Abstract] OR "microbial natural products" [Title/Abstract] OR "natural compounds" [Title/Abstract] OR "bioactive compounds" [Title/Abstract] OR "secondary metabolites" [Title/Abstract] OR "phytoconstituent" [Title/Abstract] OR "phenolic" [Title/Abstract] OR "phenolics" [Title/Abstract] OR "polyphenolic" [Title/Abstract] OR "polyphenolics" [Title/Abstract] OR "phenols" [Title/Abstract] OR "polyphenols" [Title/Abstract] OR "terpene" [Title/Abstract] OR "terpenes" [Title/Abstract] OR "alkaloid" [Title/Abstract] OR "alkaloids" [Title/Abstract] OR "Lignin" [Title/Abstract] OR "Lignins" [Title/Abstract] OR "coumarine" [Title/Abstract] OR "coumarines" [Title/Abstract] OR "sterol" [Title/Abstract] OR "Phytosterol" [Title/Abstract] OR "sterols" [Title/Abstract] OR "glycoside" [Title/Abstract] OR "glycosides" [Title/Abstract] OR "saponin" [Title/Abstract] OR "saponins" [Title/Abstract] OR "flavonoid" [Title/Abstract] OR "flavonoids" [Title/Abstract] OR "flavone" [Title/Abstract] OR "flavones" [Title/Abstract] OR "flavanol" [Title/Abstract] OR "flavanols" [Title/Abstract] OR "flavonol" [Title/Abstract] OR "flavonols" [Title/Abstract] OR "flavane" [Title/Abstract] OR "flavanes" [Title/Abstract] OR "isoflavone" [Title/Abstract] OR "isoflavones" [Title/Abstract] OR "flavan-3-ols" [Title/Abstract] OR "anthocyanidins" [Title/Abstract] OR "quaternary ammonia" [Title/Abstract] OR "peptides" [Title/Abstract] OR "polysaccharides" [Title/Abstract]
9	7 OR 8
10	3 AND 6 AND 9
11	TS=(rat OR rats OR mouse OR mice OR rodent OR animal OR animals OR preclinical studies)
12	TS=(premature ovarian failure OR premature ovary failure OR POF OR primary ovarian insufficiency OR primary ovary insufficiency

	OR POI OR ovar* and (failure or function or damage))
13	TS=(natural product OR natural products OR plant natural products OR marine natural products OR microbial natural products OR natural compounds OR bioactive compounds OR secondary metabolites OR phytoconstituent OR phenolic OR phenolics OR polyphenolic OR polyphenolics OR phenols OR polyphenols OR terpene OR terpenes OR alkaloid OR alkaloids OR Lignin OR Lignins OR coumarine OR coumarines OR sterol OR phytosterol OR sterols OR glycoside OR glycosides OR saponin OR saponins OR flavonoid OR flavonoids OR flavone OR flavones OR flavanol OR flavanols OR flavonol OR flavonols OR flavane OR “flavanes” OR “isoflavone” OR isoflavones OR flavan-3-ols OR anthocyanidins OR quaternary ammonia OR peptides OR polysaccharides)
14	11 AND 12 AND 13
15	TITLE-ABS-KEY (rat OR rats OR mouse OR mice OR rodent OR animal OR animals OR preclinical studies)
16	TITLE-ABS-KEY (premature ovarian failure OR premature ovary failure OR POF OR ovar* and (failure or function or damage))
17	TITLE-ABS-KEY (natural product OR natural products OR plant natural products OR marine natural products OR microbial natural products OR natural compounds OR bioactive compounds OR secondary metabolites OR phytoconstituent OR phenolic OR phenolics OR polyphenolic OR polyphenolics OR phenols OR polyphenols OR terpene OR terpenes OR alkaloid OR alkaloids OR Lignin OR Lignins OR coumarine OR coumarines OR sterol OR phytosterol OR sterols OR glycoside OR glycosides OR saponin OR saponins OR flavonoid OR flavonoids OR flavone OR flavones OR flavanol OR flavanols OR flavonol OR flavonols OR flavane OR “flavanes” OR “isoflavone” OR isoflavones OR flavan-3-ols OR anthocyanidins OR quaternary ammonia OR peptides OR polysaccharides)
18	15 AND 16 AND 17

2. Supplemental Figures

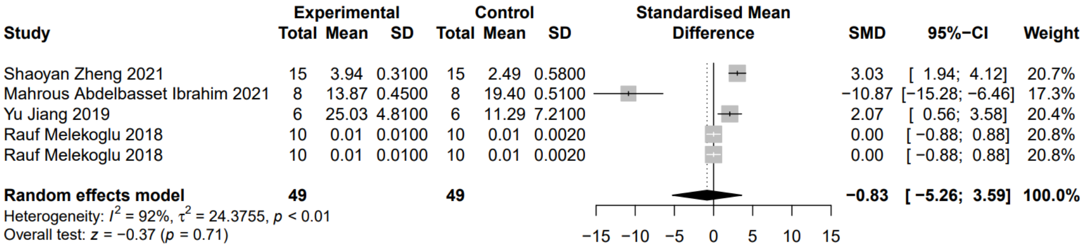

Figure S1.1. Forest plots: effects of various natural products on the secondary outcome of CAT level.

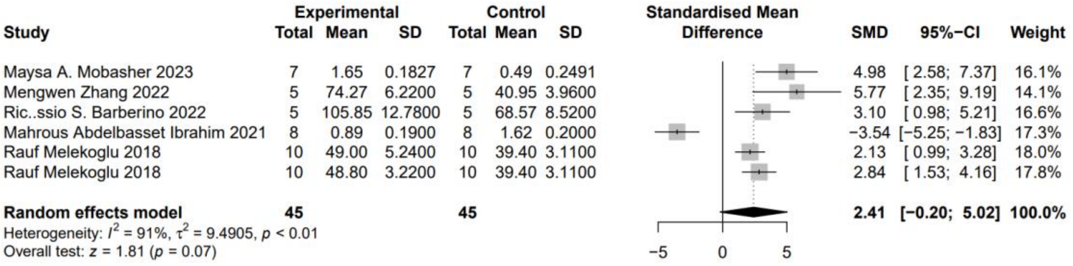

Figure S1.2. Forest plots: effects of various natural products on the secondary outcome of GSH level.

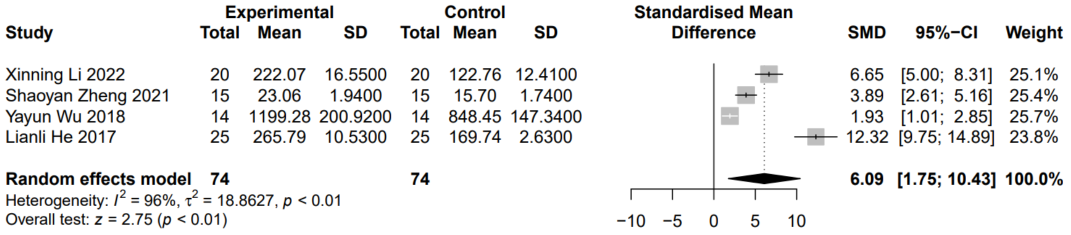

Figure S1.3. Forest plots: effects of various natural products on the secondary outcome of GSH-Px level.

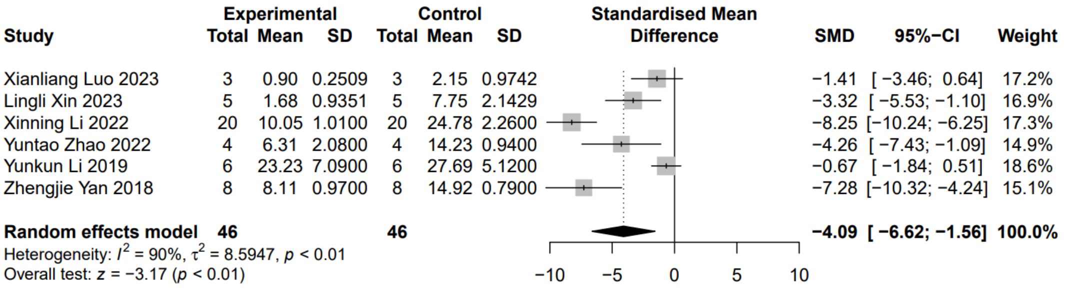

Figure S1.4. Forest plots: effects of various natural products on the secondary outcome

of GC's apoptosis.

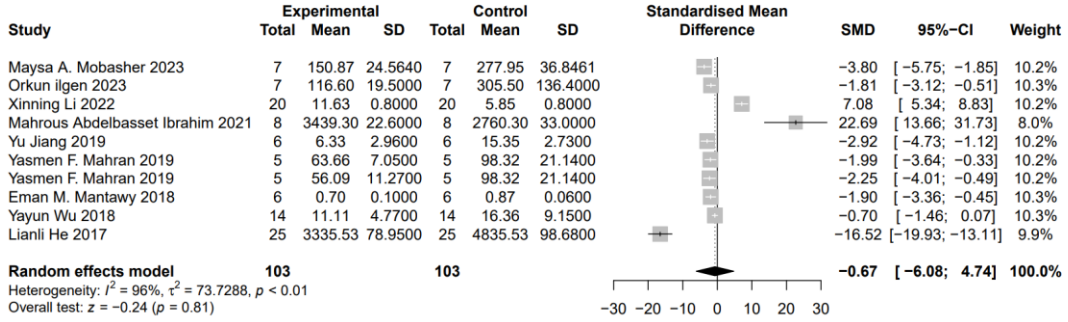

Figure S1.5. Forest plots: effects of various natural products on the secondary outcome of TNF-α level.

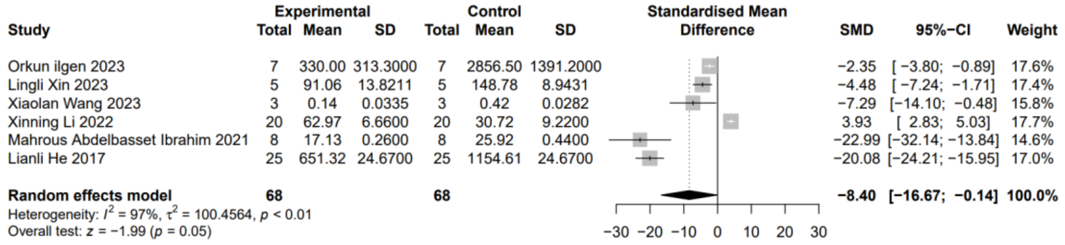

Figure S1.6. Forest plots: effects of various natural products on the secondary outcome of IL-β level.

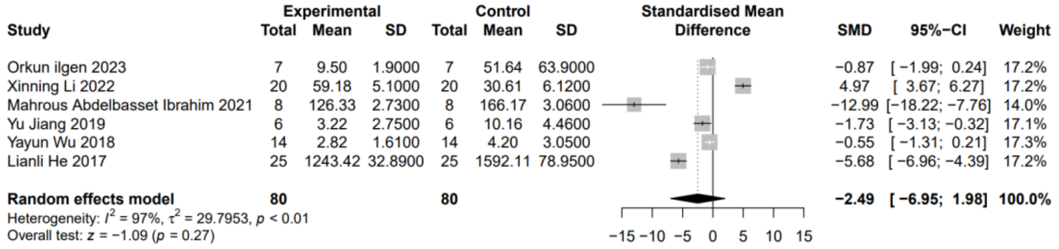

Figure S1.7. Forest plots: effects of various natural products on the secondary outcome of IL-6 level.

A

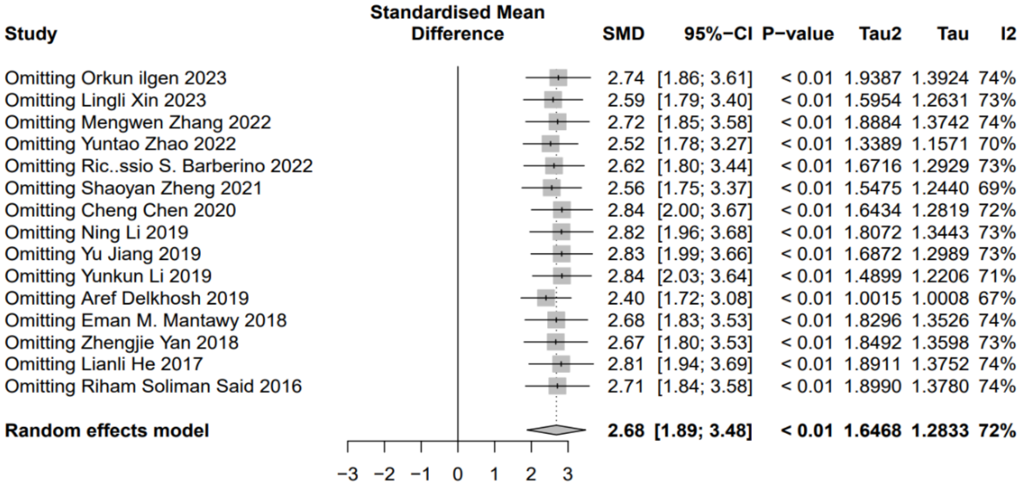
Study
Standardised Mean Difference
SMD 95%−CI P−value Tau2 Tau I2
Omitting Orkun ilgen 2023 2.74 [1.86; 3.61] < 0.01 1.9387 1.3924 74%
Omitting Lingli Xin 2023 2.59 [1.79; 3.40] < 0.01 1.5954 1.2631 73%
Omitting Mengwen Zhang 2022 2.72 [1.85; 3.58] < 0.01 1.8884 1.3742 74%
Omitting Yuntao Zhao 2022 2.52 [1.78; 3.27] < 0.01 1.3389 1.1571 70%
Omitting Ric..ssio S. Barberino 2022 2.62 [1.80; 3.44] < 0.01 1.6716 1.2929 73%
Omitting Shaoyan Zheng 2021 2.56 [1.75; 3.37] < 0.01 1.5475 1.2440 69%
Omitting Cheng Chen 2020 2.84 [2.00; 3.67] < 0.01 1.6434 1.2819 72%
Omitting Ning Li 2019 2.82 [1.96; 3.68] < 0.01 1.8072 1.3443 73%
Omitting Yu Jiang 2019 2.83 [1.99; 3.66] < 0.01 1.6872 1.2989 73%
Omitting Yunkun Li 2019 2.84 [2.03; 3.64] < 0.01 1.4899 1.2206 71%
Omitting Aref Delkhosh 2019 2.40 [1.72; 3.08] < 0.01 1.0015 1.0008 67%
Omitting Eman M. Mantawy 2018 2.68 [1.83; 3.53] < 0.01 1.8296 1.3526 74%
Omitting Zhengjie Yan 2018 2.67 [1.80; 3.53] < 0.01 1.8492 1.3598 73%
Omitting Lianli He 2017 2.81 [1.94; 3.69] < 0.01 1.8911 1.3752 74%
Omitting Riham Soliman Said 2016 2.71 [1.84; 3.58] < 0.01 1.8990 1.3780 74%
Random effects model 2.68 [1.89; 3.48] < 0.01 1.6468 1.2833 72%
−3 −2 −1 0 1 2 3

B

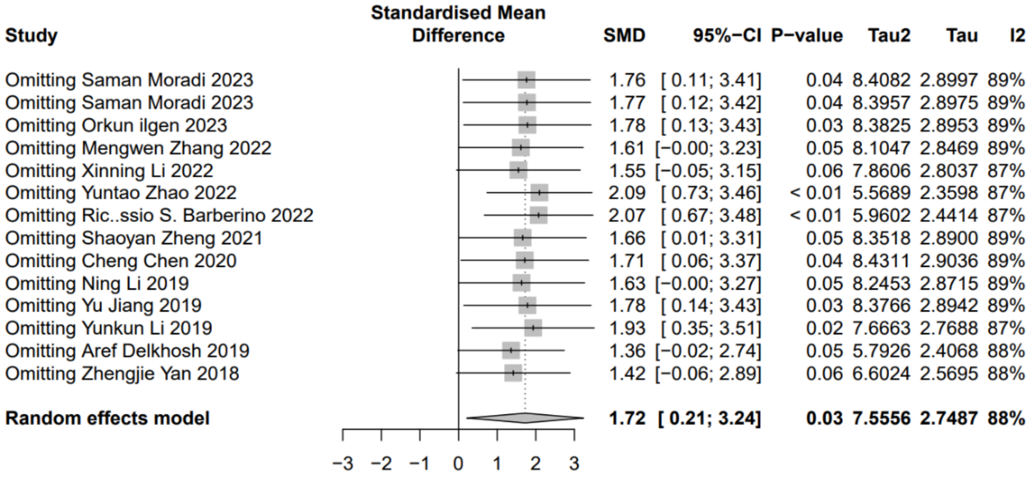
Study
Standardised Mean Difference
SMD 95%−CI P−value Tau2 Tau I2
Omitting Saman Moradi 2023 1.76 [0.11; 3.41] 0.04 8.4082 2.8997 89%
Omitting Saman Moradi 2023 1.77 [0.12; 3.42] 0.04 8.3957 2.8975 89%
Omitting Orkun ilgen 2023 1.78 [0.13; 3.43] 0.03 8.3825 2.8953 89%
Omitting Mengwen Zhang 2022 1.61 [−0.00; 3.23] 0.05 8.1047 2.8469 89%
Omitting Xinning Li 2022 1.55 [−0.05; 3.15] 0.06 7.8606 2.8037 87%
Omitting Yuntao Zhao 2022 2.09 [0.73; 3.46] < 0.01 5.5689 2.3598 87%
Omitting Ric..ssio S. Barberino 2022 2.07 [0.67; 3.48] < 0.01 5.9602 2.4414 87%
Omitting Shaoyan Zheng 2021 1.66 [0.01; 3.31] 0.05 8.3518 2.8900 89%
Omitting Cheng Chen 2020 1.71 [0.06; 3.37] 0.04 8.4311 2.9036 89%
Omitting Ning Li 2019 1.63 [−0.00; 3.27] 0.05 8.2453 2.8715 89%
Omitting Yu Jiang 2019 1.78 [0.14; 3.43] 0.03 8.3766 2.8942 89%
Omitting Yunkun Li 2019 1.93 [0.35; 3.51] 0.02 7.6663 2.7688 87%
Omitting Aref Delkhosh 2019 1.36 [−0.02; 2.74] 0.05 5.7926 2.4068 88%
Omitting Zhengjie Yan 2018 1.42 [−0.06; 2.89] 0.06 6.6024 2.5695 88%
Random effects model 1.72 [0.21; 3.24] 0.03 7.5556 2.7487 88%
−3 −2 −1 0 1 2 3

C

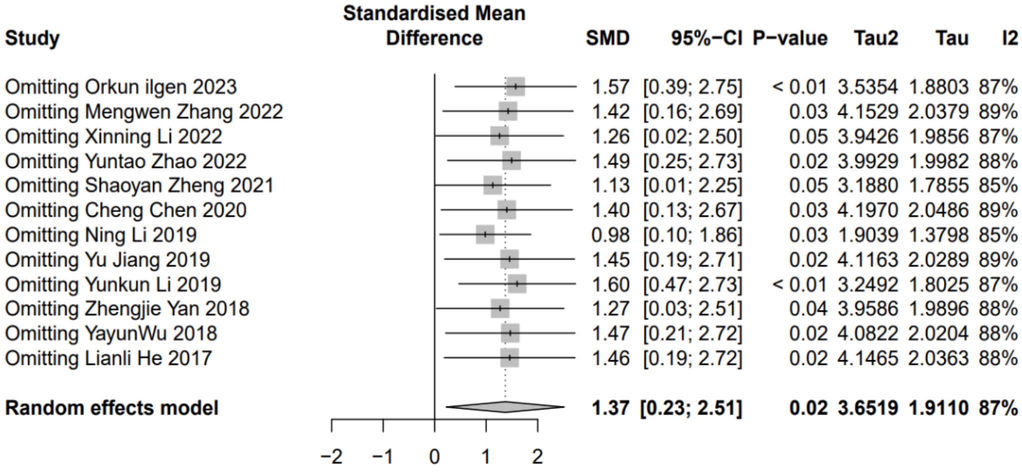
Study
Standardised Mean Difference
SMD 95%−CI P−value Tau2 Tau I2
Omitting Orkun ilgen 2023 1.57 [0.39; 2.75] < 0.01 3.5354 1.8803 87%
Omitting Mengwen Zhang 2022 1.42 [0.16; 2.69] 0.03 4.1529 2.0379 89%
Omitting Xinning Li 2022 1.26 [0.02; 2.50] 0.05 3.9426 1.9856 87%
Omitting Yuntao Zhao 2022 1.49 [0.25; 2.73] 0.02 3.9929 1.9982 88%
Omitting Shaoyan Zheng 2021 1.13 [0.01; 2.25] 0.05 3.1880 1.7855 85%
Omitting Cheng Chen 2020 1.40 [0.13; 2.67] 0.03 4.1970 2.0486 89%
Omitting Ning Li 2019 0.98 [0.10; 1.86] 0.03 1.9039 1.3798 85%
Omitting Yu Jiang 2019 1.45 [0.19; 2.71] 0.02 4.1163 2.0289 89%
Omitting Yunkun Li 2019 1.60 [0.47; 2.73] < 0.01 3.2492 1.8025 87%
Omitting Zhengjie Yan 2018 1.27 [0.03; 2.51] 0.04 3.9586 1.9896 88%
Omitting YayunWu 2018 1.47 [0.21; 2.72] 0.02 4.0822 2.0204 88%
Omitting Lianli He 2017 1.46 [0.19; 2.72] 0.02 4.1465 2.0363 88%
Random effects model 1.37 [0.23; 2.51] 0.02 3.6519 1.9110 87%
−2 −1 0 1 2

D

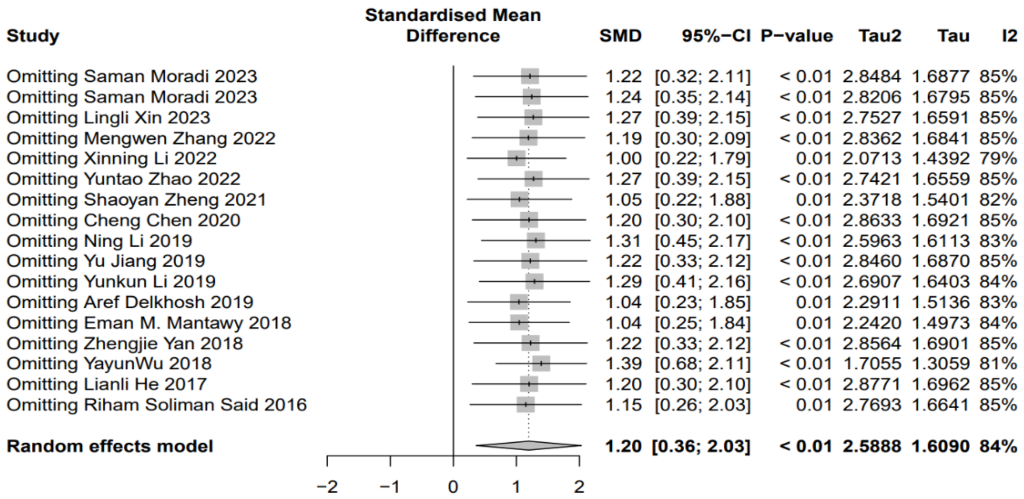

E

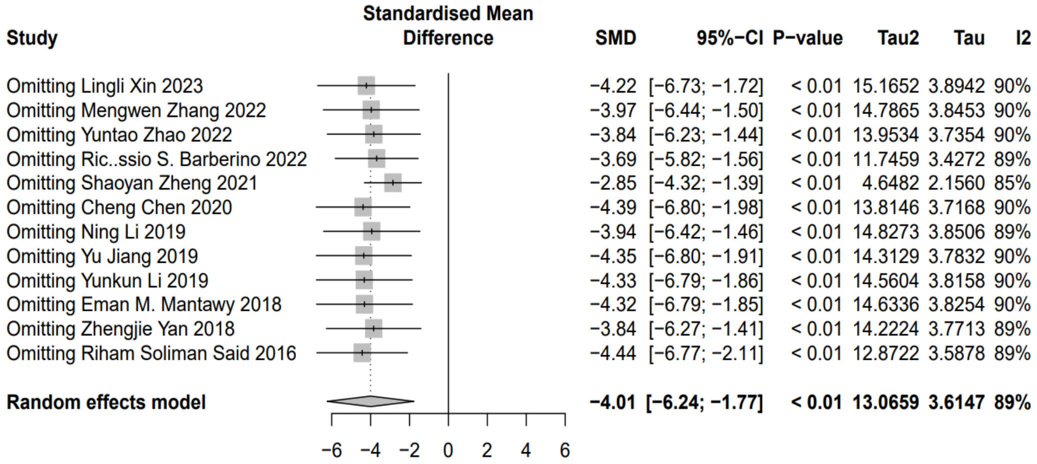

Figure S2.1. Sensitivity analysis of various natural products on the primary outcomes of follicular development. (A) counts of primordial follicles, (B) counts of primary follicles, (C) counts of secondary follicles, (D) counts of antral follicles, (E) counts of atretic follicles.

A

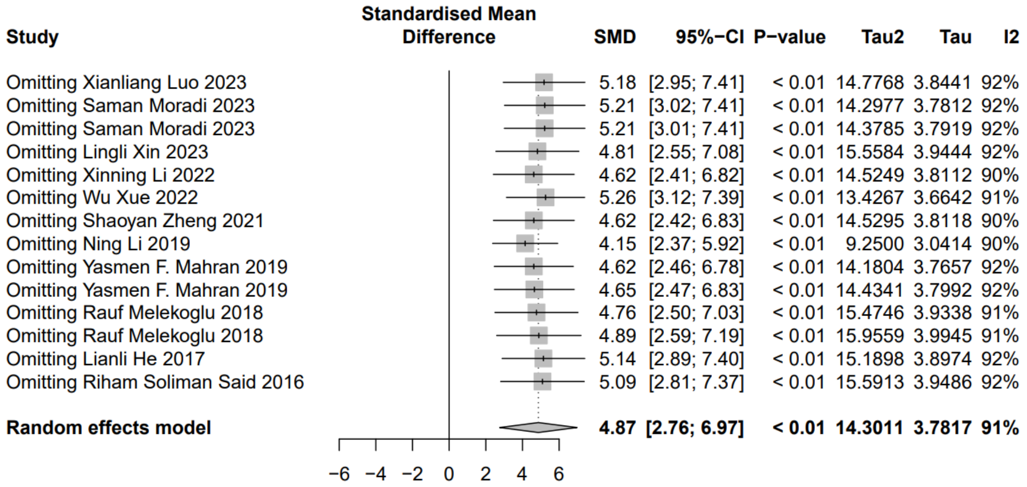
Standardised Mean Difference
Study
SMD
95%−CI
P−value
Tau2
Tau
I2
Omitting Xianliang Luo 2023 5.18 [2.95; 7.41] < 0.01 14.7768 3.8441 92%
Omitting Saman Moradi 2023 5.21 [3.02; 7.41] < 0.01 14.2977 3.7812 92%
Omitting Saman Moradi 2023 5.21 [3.01; 7.41] < 0.01 14.3785 3.7919 92%
Omitting Lingli Xin 2023 4.81 [2.55; 7.08] < 0.01 15.5584 3.9444 92%
Omitting Xinning Li 2022 4.62 [2.41; 6.82] < 0.01 14.5249 3.8112 90%
Omitting Wu Xue 2022 5.26 [3.12; 7.39] < 0.01 13.4267 3.6642 91%
Omitting Shaoyan Zheng 2021 4.62 [2.42; 6.83] < 0.01 14.5295 3.8118 90%
Omitting Ning Li 2019 4.15 [2.37; 5.92] < 0.01 9.2500 3.0414 90%
Omitting Yasmen F. Mahran 2019 4.62 [2.46; 6.78] < 0.01 14.1804 3.7657 92%
Omitting Yasmen F. Mahran 2019 4.65 [2.47; 6.83] < 0.01 14.4341 3.7992 92%
Omitting Rauf Melekoglu 2018 4.76 [2.50; 7.03] < 0.01 15.4746 3.9338 91%
Omitting Rauf Melekoglu 2018 4.89 [2.59; 7.19] < 0.01 15.9559 3.9945 91%
Omitting Lianli He 2017 5.14 [2.89; 7.40] < 0.01 15.1898 3.8974 92%
Omitting Riham Soliman Said 2016 5.09 [2.81; 7.37] < 0.01 15.5913 3.9486 92%
Random effects model 4.87 [2.76; 6.97] < 0.01 14.3011 3.7817 91%
−6 −4 −2 0 2 4 6

B

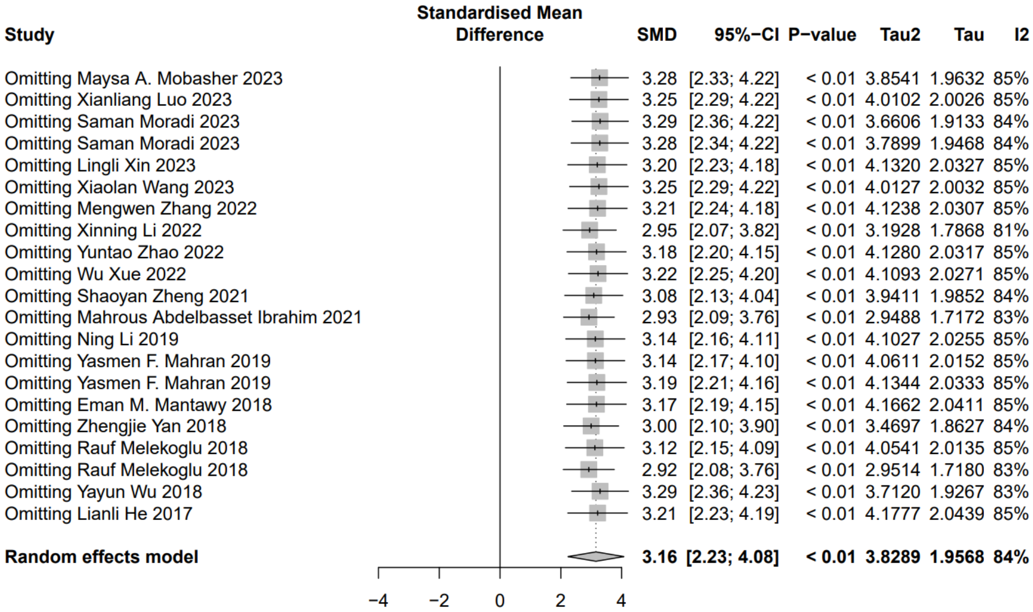
Standardised Mean Difference
Study
SMD
95%−CI
P−value
Tau2
Tau
I2
Omitting Maysa A. Mobasher 2023 3.28 [2.33; 4.22] < 0.01 3.8541 1.9632 85%
Omitting Xianliang Luo 2023 3.25 [2.29; 4.22] < 0.01 4.0102 2.0026 85%
Omitting Saman Moradi 2023 3.29 [2.36; 4.22] < 0.01 3.6606 1.9133 84%
Omitting Saman Moradi 2023 3.28 [2.34; 4.22] < 0.01 3.7899 1.9468 84%
Omitting Lingli Xin 2023 3.20 [2.23; 4.18] < 0.01 4.1320 2.0327 85%
Omitting Xiaolan Wang 2023 3.25 [2.29; 4.22] < 0.01 4.0127 2.0032 85%
Omitting Mengwen Zhang 2022 3.21 [2.24; 4.18] < 0.01 4.1238 2.0307 85%
Omitting Xinning Li 2022 2.95 [2.07; 3.82] < 0.01 3.1928 1.7868 81%
Omitting Yuntao Zhao 2022 3.18 [2.20; 4.15] < 0.01 4.1280 2.0317 85%
Omitting Wu Xue 2022 3.22 [2.25; 4.20] < 0.01 4.1093 2.0271 85%
Omitting Shaoyan Zheng 2021 3.08 [2.13; 4.04] < 0.01 3.9411 1.9852 84%
Omitting Mahrous Abdelbasset Ibrahim 2021 2.93 [2.09; 3.76] < 0.01 2.9488 1.7172 83%
Omitting Ning Li 2019 3.14 [2.16; 4.11] < 0.01 4.1027 2.0255 85%
Omitting Yasmen F. Mahran 2019 3.14 [2.17; 4.10] < 0.01 4.0611 2.0152 85%
Omitting Yasmen F. Mahran 2019 3.19 [2.21; 4.16] < 0.01 4.1344 2.0333 85%
Omitting Eman M. Mantawy 2018 3.17 [2.19; 4.15] < 0.01 4.1662 2.0411 85%
Omitting Zhengjie Yan 2018 3.00 [2.10; 3.90] < 0.01 3.4697 1.8627 84%
Omitting Rauf Melekoglu 2018 3.12 [2.15; 4.09] < 0.01 4.0541 2.0135 85%
Omitting Rauf Melekoglu 2018 2.92 [2.08; 3.76] < 0.01 2.9514 1.7180 83%
Omitting Yayun Wu 2018 3.29 [2.36; 4.23] < 0.01 3.7120 1.9267 83%
Omitting Lianli He 2017 3.21 [2.23; 4.19] < 0.01 4.1777 2.0439 85%
Random effects model 3.16 [2.23; 4.08] < 0.01 3.8289 1.9568 84%
−4 −2 0 2 4

C

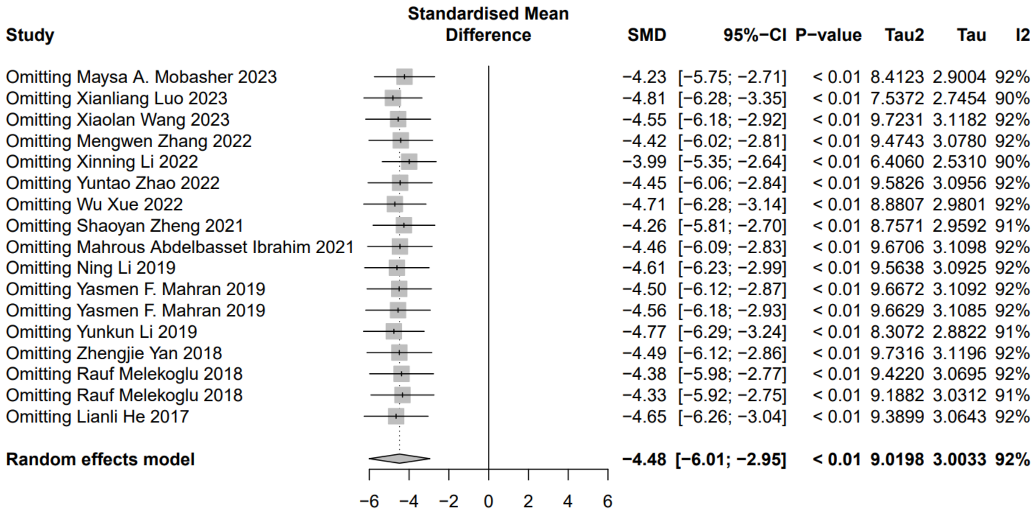
Standardised Mean Difference
Study
SMD
95%−CI
P−value
Tau2
Tau
I2
Omitting Maysa A. Mobasher 2023 −4.23 [−5.75; −2.71] < 0.01 8.4123 2.9004 92%
Omitting Xianliang Luo 2023 −4.81 [−6.28; −3.35] < 0.01 7.5372 2.7454 90%
Omitting Xiaolan Wang 2023 −4.55 [−6.18; −2.92] < 0.01 9.7231 3.1182 92%
Omitting Mengwen Zhang 2022 −4.42 [−6.02; −2.81] < 0.01 9.4743 3.0780 92%
Omitting Xinning Li 2022 −3.99 [−5.35; −2.64] < 0.01 6.4060 2.5310 90%
Omitting Yuntao Zhao 2022 −4.45 [−6.06; −2.84] < 0.01 9.5826 3.0956 92%
Omitting Wu Xue 2022 −4.71 [−6.28; −3.14] < 0.01 8.8807 2.9801 92%
Omitting Shaoyan Zheng 2021 −4.26 [−5.81; −2.70] < 0.01 8.7571 2.9592 91%
Omitting Mahrous Abdelbasset Ibrahim 2021 −4.46 [−6.09; −2.83] < 0.01 9.6706 3.1098 92%
Omitting Ning Li 2019 −4.61 [−6.23; −2.99] < 0.01 9.5638 3.0925 92%
Omitting Yasmen F. Mahran 2019 −4.50 [−6.12; −2.87] < 0.01 9.6672 3.1092 92%
Omitting Yasmen F. Mahran 2019 −4.56 [−6.18; −2.93] < 0.01 9.6629 3.1085 92%
Omitting Yunkun Li 2019 −4.77 [−6.29; −3.24] < 0.01 8.3072 2.8822 91%
Omitting Zhengjie Yan 2018 −4.49 [−6.12; −2.86] < 0.01 9.7316 3.1196 92%
Omitting Rauf Melekoglu 2018 −4.38 [−5.98; −2.77] < 0.01 9.4220 3.0695 92%
Omitting Rauf Melekoglu 2018 −4.33 [−5.92; −2.75] < 0.01 9.1882 3.0312 91%
Omitting Lianli He 2017 −4.65 [−6.26; −3.04] < 0.01 9.3899 3.0643 92%
Random effects model −4.48 [−6.01; −2.95] < 0.01 9.0198 3.0033 92%
−6 −4 −2 0 2 4 6

D

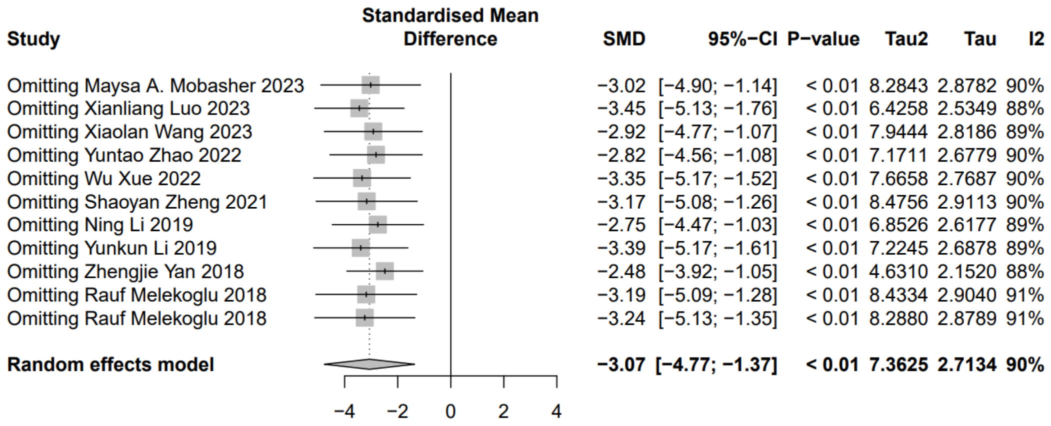

E

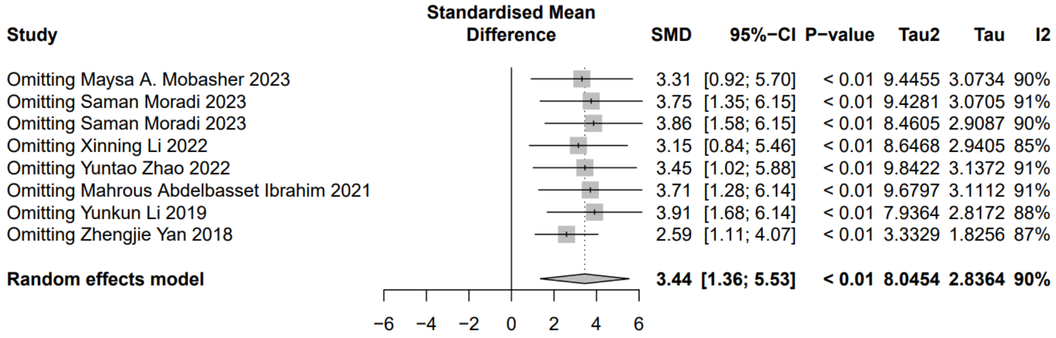

Figure S2.2. Sensitivity analysis of various natural products on the primary outcomes of ovarian function. (A) AMH, (B) E2, (C) FSH, (D) LH, (E) P.

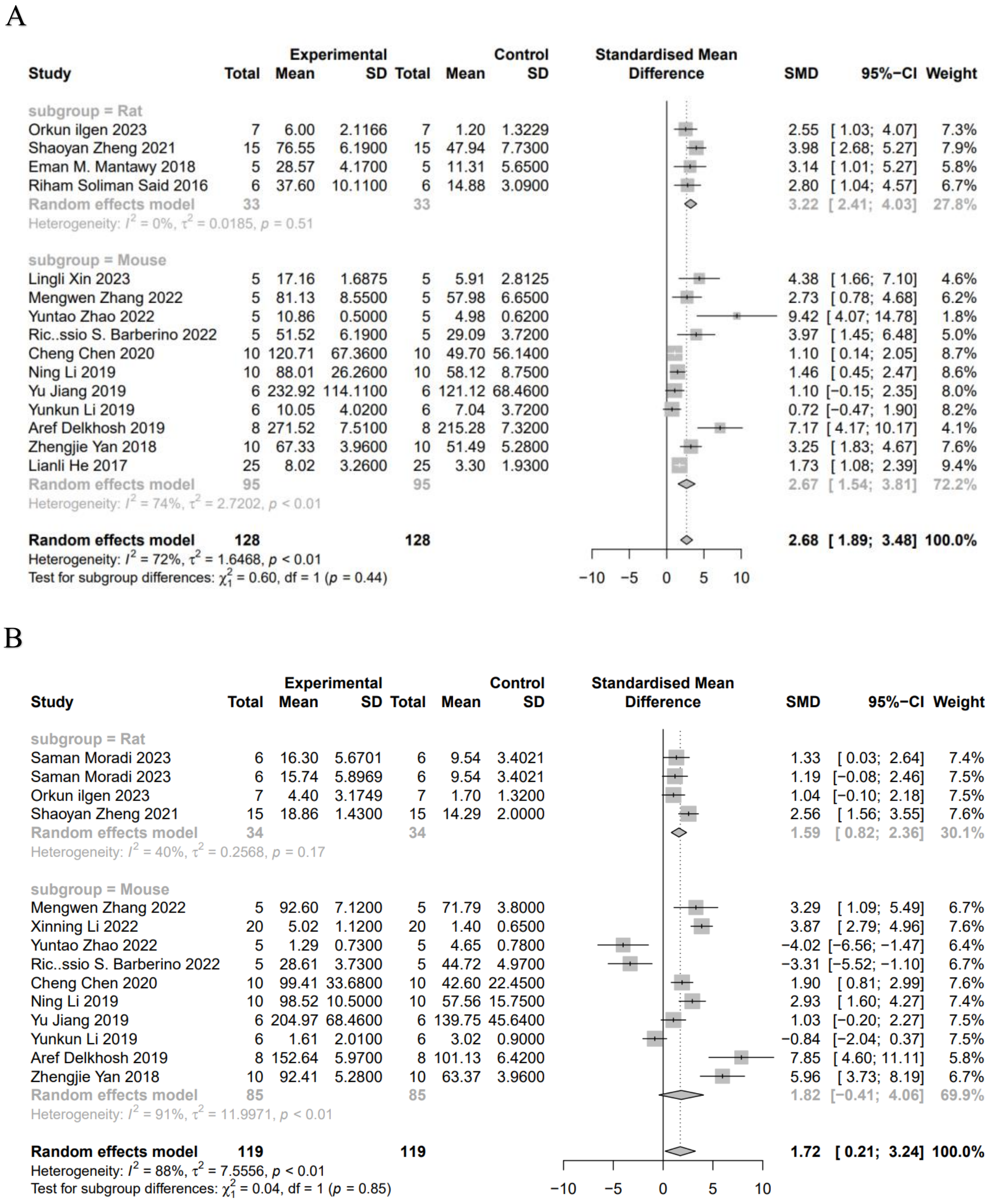
A
Study | Experimental Total | Mean | SD | Control Total | Mean | SD | Standardised Mean Difference | SMD | 95%−CI | Weight
subgroup = Rat
Orkun ilgen 2023 7 6.00 2.1166 7 1.20 1.3229 2.55 [1.03; 4.07] 7.3%
Shaoyan Zheng 2021 15 76.55 6.1900 15 47.94 7.7300 3.98 [2.68; 5.27] 7.9%
Eman M. Mantawy 2018 5 28.57 4.1700 5 11.31 5.6500 3.14 [1.01; 5.27] 5.8%
Riham Soliman Said 2016 6 37.60 10.1100 6 14.88 3.0900 2.80 [1.04; 4.57] 6.7%
Random effects model 33 33 3.22 [2.41; 4.03] 27.8%
Heterogeneity: I^2 = 0%, τ^2 = 0.0185, p = 0.51
subgroup = Mouse
Lingli Xin 2023 5 17.16 1.6875 5 5.91 2.8125 4.38 [1.66; 7.10] 4.6%
Mengwen Zhang 2022 5 81.13 8.5500 5 57.98 6.6500 2.73 [0.78; 4.68] 6.2%
Yuntao Zhao 2022 5 10.86 0.5000 5 4.98 0.6200 9.42 [4.07; 14.78] 1.8%
Ric..ssio S. Barberino 2022 5 51.52 6.1900 5 29.09 3.7200 3.97 [1.45; 6.48] 5.0%
Cheng Chen 2020 10 120.71 67.3600 10 49.70 56.1400 1.10 [0.14; 2.05] 8.7%
Ning Li 2019 10 88.01 26.2600 10 58.12 8.7500 1.46 [0.45; 2.47] 8.6%
Yu Jiang 2019 6 232.92 114.1100 6 121.12 68.4600 1.10 [−0.15; 2.35] 8.0%
Yunkun Li 2019 6 10.05 4.0200 6 7.04 3.7200 0.72 [−0.47; 1.90] 8.2%
Aref Delkhosh 2019 8 271.52 7.5100 8 215.28 7.3200 7.17 [4.17; 10.17] 4.1%
Zhengjie Yan 2018 10 67.33 3.9600 10 51.49 5.2800 3.25 [1.83; 4.67] 7.6%
Lianli He 2017 25 8.02 3.2600 25 3.30 1.9300 1.73 [1.08; 2.39] 9.4%
Random effects model 95 95 2.67 [1.54; 3.81] 72.2%
Heterogeneity: I^2 = 74%, τ^2 = 2.7202, p < 0.01
Random effects model 128 128 2.68 [1.89; 3.48] 100.0%
Heterogeneity: I^2 = 72%, τ^2 = 1.6468, p < 0.01
Test for subgroup differences: χ^2_1 = 0.60, df = 1 (p = 0.44)
−10 −5 0 5 10
B
Study | Experimental Total | Mean | SD | Control Total | Mean | SD | Standardised Mean Difference | SMD | 95%−CI | Weight
subgroup = Rat
Saman Moradi 2023 6 16.30 5.6701 6 9.54 3.4021 1.33 [0.03; 2.64] 7.4%
Saman Moradi 2023 6 15.74 5.8969 6 9.54 3.4021 1.19 [−0.08; 2.46] 7.5%
Orkun ilgen 2023 7 4.40 3.1749 7 1.70 1.3200 1.04 [−0.10; 2.18] 7.5%
Shaoyan Zheng 2021 15 18.86 1.4300 15 14.29 2.0000 2.56 [1.56; 3.55] 7.6%
Random effects model 34 34 1.59 [0.82; 2.36] 30.1%
Heterogeneity: I^2 = 40%, τ^2 = 0.2568, p = 0.17
subgroup = Mouse
Mengwen Zhang 2022 5 92.60 7.1200 5 71.79 3.8000 3.29 [1.09; 5.49] 6.7%
Xinning Li 2022 20 5.02 1.1200 20 1.40 0.6500 3.87 [2.79; 4.96] 7.6%
Yuntao Zhao 2022 5 1.29 0.7300 5 4.65 0.7800 −4.02 [−6.56; −1.47] 6.4%
Ric..ssio S. Barberino 2022 5 28.61 3.7300 5 44.72 4.9700 −3.31 [−5.52; −1.10] 6.7%
Cheng Chen 2020 10 99.41 33.6800 10 42.60 22.4500 1.90 [0.81; 2.99] 7.6%
Ning Li 2019 10 98.52 10.5000 10 57.56 15.7500 2.93 [1.60; 4.27] 7.4%
Yu Jiang 2019 6 204.97 68.4600 6 139.75 45.6400 1.03 [−0.20; 2.27] 7.5%
Yunkun Li 2019 6 1.61 2.0100 6 3.02 0.9000 −0.84 [−2.04; 0.37] 7.5%
Aref Delkhosh 2019 8 152.64 5.9700 8 101.13 6.4200 7.85 [4.60; 11.11] 5.8%
Zhengjie Yan 2018 10 92.41 5.2800 10 63.37 3.9600 5.96 [3.73; 8.19] 6.7%
Random effects model 85 85 1.82 [−0.41; 4.06] 69.9%
Heterogeneity: I^2 = 91%, τ^2 = 11.9971, p < 0.01
Random effects model 119 119 1.72 [0.21; 3.24] 100.0%
Heterogeneity: I^2 = 88%, τ^2 = 7.5556, p < 0.01
Test for subgroup differences: χ^2_1 = 0.04, df = 1 (p = 0.85)
−10 −5 0 5 10

C

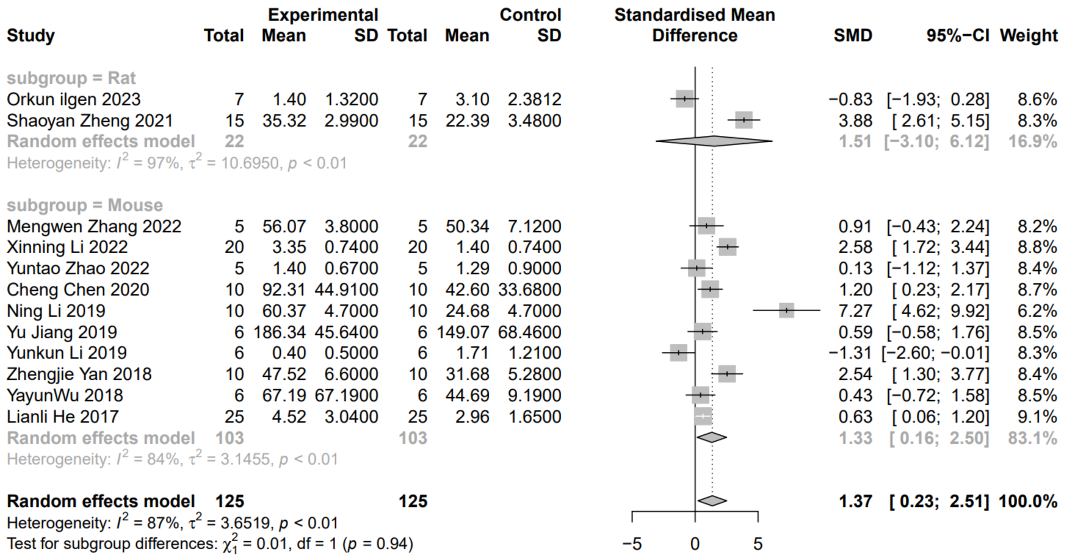

Study | Experimental Total Mean SD | Control Total Mean SD | Standardised Mean Difference | SMD | 95%−CI | Weight
subgroup = Rat
Orkun ilgen 2023 7 1.40 1.3200 7 3.10 2.3812 −0.83 [−1.93; 0.28] 8.6%
Shaoyan Zheng 2021 15 35.32 2.9900 15 22.39 3.4800 3.88 [2.61; 5.15] 8.3%
Random effects model 22 22 1.51 [−3.10; 6.12] 16.9%
Heterogeneity: I^2 = 97%, τ^2 = 10.6950, p < 0.01
subgroup = Mouse
Mengwen Zhang 2022 5 56.07 3.8000 5 50.34 7.1200 0.91 [−0.43; 2.24] 8.2%
Xinning Li 2022 20 3.35 0.7400 20 1.40 0.7400 2.58 [1.72; 3.44] 8.8%
Yuntao Zhao 2022 5 1.40 0.6700 5 1.29 0.9000 0.13 [−1.12; 1.37] 8.4%
Cheng Chen 2020 10 92.31 44.9100 10 42.60 33.6800 1.20 [0.23; 2.17] 8.7%
Ning Li 2019 10 60.37 4.7000 10 24.68 4.7000 7.27 [4.62; 9.92] 6.2%
Yu Jiang 2019 6 186.34 45.6400 6 149.07 68.4600 0.59 [−0.58; 1.76] 8.5%
Yunkun Li 2019 6 0.40 0.5000 6 1.71 1.2100 −1.31 [−2.60; −0.01] 8.3%
Zhengjie Yan 2018 10 47.52 6.6000 10 31.68 5.2800 2.54 [1.30; 3.77] 8.4%
YayunWu 2018 6 67.19 67.1900 6 44.69 9.1900 0.43 [−0.72; 1.58] 8.5%
Lianli He 2017 25 4.52 3.0400 25 2.96 1.6500 0.63 [0.06; 1.20] 9.1%
Random effects model 103 103 1.33 [0.16; 2.50] 83.1%
Heterogeneity: I^2 = 84%, τ^2 = 3.1455, p < 0.01
Random effects model 125 125 1.37 [0.23; 2.51] 100.0%
Heterogeneity: I^2 = 87%, τ^2 = 3.6519, p < 0.01
Test for subgroup differences: χ^2_1 = 0.01, df = 1 (p = 0.94)
−5
0
5

D

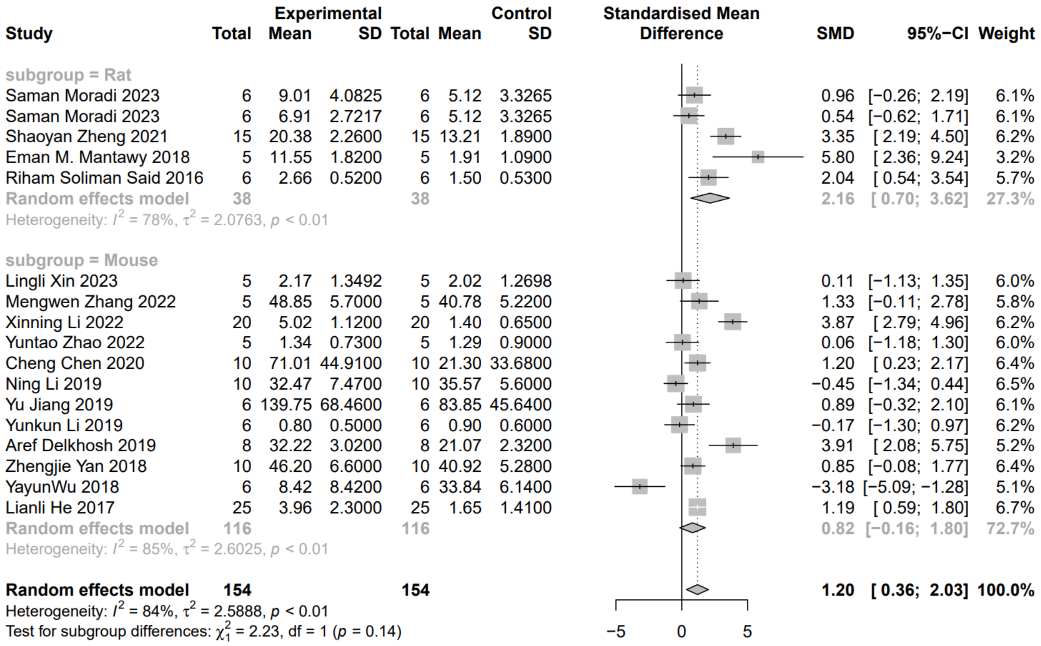

Study | Experimental Total Mean SD | Control Total Mean SD | Standardised Mean Difference | SMD | 95%−CI | Weight
subgroup = Rat
Saman Moradi 2023 6 9.01 4.0825 6 5.12 3.3265 0.96 [−0.26; 2.19] 6.1%
Saman Moradi 2023 6 6.91 2.7217 6 5.12 3.3265 0.54 [−0.62; 1.71] 6.1%
Shaoyan Zheng 2021 15 20.38 2.2600 15 13.21 1.8900 3.35 [2.19; 4.50] 6.2%
Eman M. Mantawy 2018 5 11.55 1.8200 5 1.91 1.0900 5.80 [2.36; 9.24] 3.2%
Riham Soliman Said 2016 6 2.66 0.5200 6 1.50 0.5300 2.04 [0.54; 3.54] 5.7%
Random effects model 38 38 2.16 [0.70; 3.62] 27.3%
Heterogeneity: I^2 = 78%, τ^2 = 2.0763, p < 0.01
subgroup = Mouse
Lingli Xin 2023 5 2.17 1.3492 5 2.02 1.2698 0.11 [−1.13; 1.35] 6.0%
Mengwen Zhang 2022 5 48.85 5.7000 5 40.78 5.2200 1.33 [−0.11; 2.78] 5.8%
Xinning Li 2022 20 5.02 1.1200 20 1.40 0.6500 3.87 [2.79; 4.96] 6.2%
Yuntao Zhao 2022 5 1.34 0.7300 5 1.29 0.9000 0.06 [−1.18; 1.30] 6.0%
Cheng Chen 2020 10 71.01 44.9100 10 21.30 33.6800 1.20 [0.23; 2.17] 6.4%
Ning Li 2019 10 32.47 7.4700 10 35.57 5.6000 −0.45 [−1.34; 0.44] 6.5%
Yu Jiang 2019 6 139.75 68.4600 6 83.85 45.6400 0.89 [−0.32; 2.10] 6.1%
Yunkun Li 2019 6 0.80 0.5000 6 0.90 0.6000 −0.17 [−1.30; 0.97] 6.2%
Aref Delkhosh 2019 8 32.22 3.0200 8 21.07 2.3200 3.91 [2.08; 5.75] 5.2%
Zhengjie Yan 2018 10 46.20 6.6000 10 40.92 5.2800 0.85 [−0.08; 1.77] 6.4%
YayunWu 2018 6 8.42 8.4200 6 33.84 6.1400 −3.18 [−5.09; −1.28] 5.1%
Lianli He 2017 25 3.96 2.3000 25 1.65 1.4100 1.19 [0.59; 1.80] 6.7%
Random effects model 116 116 0.82 [−0.16; 1.80] 72.7%
Heterogeneity: I^2 = 85%, τ^2 = 2.6025, p < 0.01
Random effects model 154 154 1.20 [0.36; 2.03] 100.0%
Heterogeneity: I^2 = 84%, τ^2 = 2.5888, p < 0.01
Test for subgroup differences: χ^2_1 = 2.23, df = 1 (p = 0.14)
−5
0
5

E

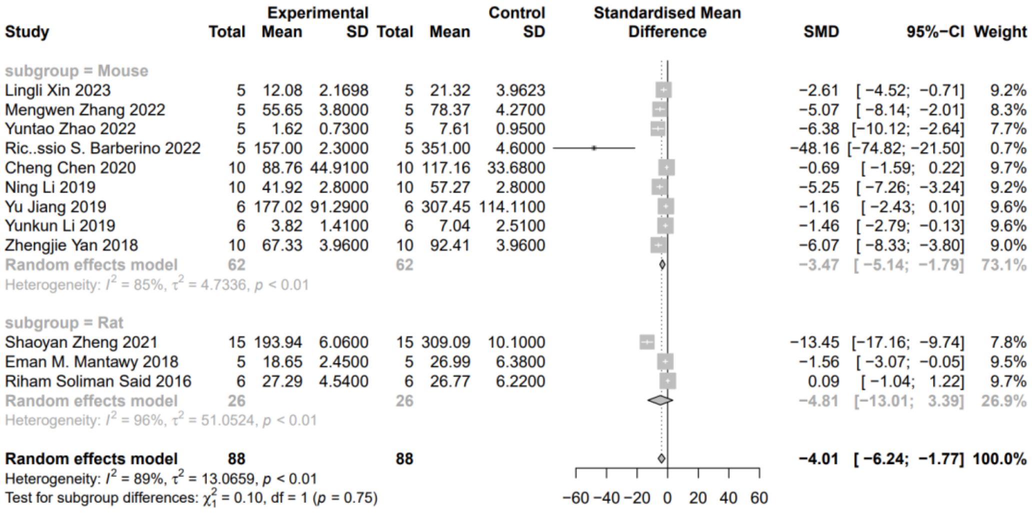
Study
Experimental Total Mean SD
Control Total Mean SD
Standardised Mean Difference
SMD 95%−CI Weight
subgroup = Mouse
Lingli Xin 2023 5 12.08 2.1698 5 21.32 3.9623 −2.61 [−4.52; −0.71] 9.2%
Mengwen Zhang 2022 5 55.65 3.8000 5 78.37 4.2700 −5.07 [−8.14; −2.01] 8.3%
Yuntao Zhao 2022 5 1.62 0.7300 5 7.61 0.9500 −6.38 [−10.12; −2.64] 7.7%
Ric..ssio S. Barberino 2022 5 157.00 2.3000 5 351.00 4.6000 −48.16 [−74.82; −21.50] 0.7%
Cheng Chen 2020 10 88.76 44.9100 10 117.16 33.6800 −0.69 [−1.59; 0.22] 9.7%
Ning Li 2019 10 41.92 2.8000 10 57.27 2.8000 −5.25 [−7.26; −3.24] 9.2%
Yu Jiang 2019 6 177.02 91.2900 6 307.45 114.1100 −1.16 [−2.43; 0.10] 9.6%
Yunkun Li 2019 6 3.82 1.4100 6 7.04 2.5100 −1.46 [−2.79; −0.13] 9.6%
Zhengjie Yan 2018 10 67.33 3.9600 10 92.41 3.9600 −6.07 [−8.33; −3.80] 9.0%
Random effects model 62 62 −3.47 [−5.14; −1.79] 73.1%
Heterogeneity: I^2 = 85%, τ^2 = 4.7336, p < 0.01
subgroup = Rat
Shaoyan Zheng 2021 15 193.94 6.0600 15 309.09 10.1000 −13.45 [−17.16; −9.74] 7.8%
Eman M. Mantawy 2018 5 18.65 2.4500 5 26.99 6.3800 −1.56 [−3.07; −0.05] 9.5%
Riham Soliman Said 2016 6 27.29 4.5400 6 26.77 6.2200 0.09 [−1.04; 1.22] 9.7%
Random effects model 26 26 −4.81 [−13.01; 3.39] 26.9%
Heterogeneity: I^2 = 96%, τ^2 = 51.0524, p < 0.01
Random effects model 88 88 −4.01 [−6.24; −1.77] 100.0%
Heterogeneity: I^2 = 89%, τ^2 = 13.0659, p < 0.01
Test for subgroup differences: χ^2_1 = 0.10, df = 1 (p = 0.75)
−60 −40 −20 0 20 40 60

F

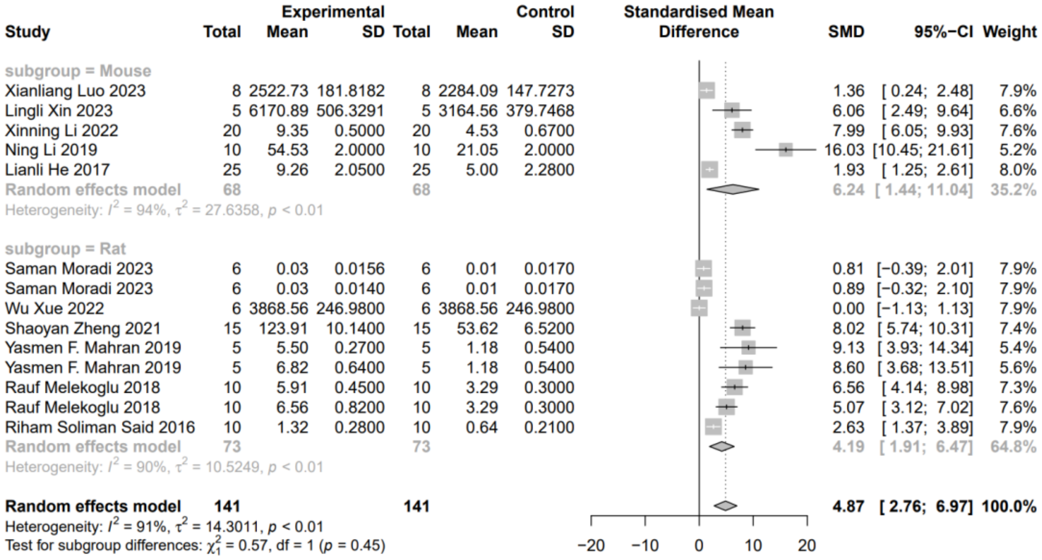
Study
Experimental Total Mean SD
Control Total Mean SD
Standardised Mean Difference
SMD 95%−CI Weight
subgroup = Mouse
Xianliang Luo 2023 8 2522.73 181.8182 8 2284.09 147.7273 1.36 [0.24; 2.48] 7.9%
Lingli Xin 2023 5 6170.89 506.3291 5 3164.56 379.7468 6.06 [2.49; 9.64] 6.6%
Xinning Li 2022 20 9.35 0.5000 20 4.53 0.6700 7.99 [6.05; 9.93] 7.6%
Ning Li 2019 10 54.53 2.0000 10 21.05 2.0000 16.03 [10.45; 21.61] 5.2%
Lianli He 2017 25 9.26 2.0500 25 5.00 2.2800 1.93 [1.25; 2.61] 8.0%
Random effects model 68 68 6.24 [1.44; 11.04] 35.2%
Heterogeneity: I^2 = 94%, τ^2 = 27.6358, p < 0.01
subgroup = Rat
Saman Moradi 2023 6 0.03 0.0156 6 0.01 0.0170 0.81 [−0.39; 2.01] 7.9%
Saman Moradi 2023 6 0.03 0.0140 6 0.01 0.0170 0.89 [−0.32; 2.10] 7.9%
Wu Xue 2022 6 3868.56 246.9800 6 3868.56 246.9800 0.00 [−1.13; 1.13] 7.9%
Shaoyan Zheng 2021 15 123.91 10.1400 15 53.62 6.5200 8.02 [5.74; 10.31] 7.4%
Yasmen F. Mahran 2019 5 5.50 0.2700 5 1.18 0.5400 9.13 [3.93; 14.34] 5.4%
Yasmen F. Mahran 2019 5 6.82 0.6400 5 1.18 0.5400 8.60 [3.68; 13.51] 5.6%
Rauf Melekoglu 2018 10 5.91 0.4500 10 3.29 0.3000 6.56 [4.14; 8.98] 7.3%
Rauf Melekoglu 2018 10 6.56 0.8200 10 3.29 0.3000 5.07 [3.12; 7.02] 7.6%
Riham Soliman Said 2016 10 1.32 0.2800 10 0.64 0.2100 2.63 [1.37; 3.89] 7.9%
Random effects model 73 73 4.19 [1.91; 6.47] 64.8%
Heterogeneity: I^2 = 90%, τ^2 = 10.5249, p < 0.01
Random effects model 141 141 4.87 [2.76; 6.97] 100.0%
Heterogeneity: I^2 = 91%, τ^2 = 14.3011, p < 0.01
Test for subgroup differences: χ^2_1 = 0.57, df = 1 (p = 0.45)
−20 −10 0 10 20

G

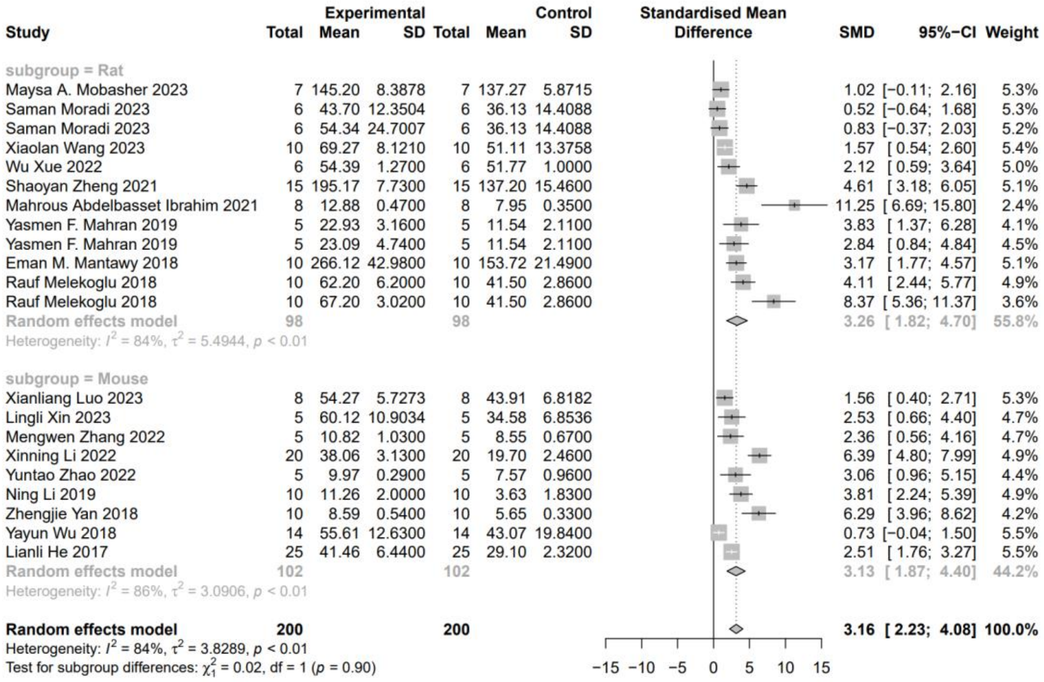

Study | Experimental Total | Mean | SD | Control Total | Mean | SD | Standardised Mean Difference | SMD | 95%-CI | Weight
subgroup = Rat
Maysa A. Mobasher 2023 7 145.20 8.3878 7 137.27 5.8715 1.02 [−0.11; 2.16] 5.3%
Saman Moradi 2023 6 43.70 12.3504 6 36.13 14.4088 0.52 [−0.64; 1.68] 5.3%
Saman Moradi 2023 6 54.34 24.7007 6 36.13 14.4088 0.83 [−0.37; 2.03] 5.2%
Xiaolan Wang 2023 10 69.27 8.1210 10 51.11 13.3758 1.57 [0.54; 2.60] 5.4%
Wu Xue 2022 6 54.39 1.2700 6 51.77 1.0000 2.12 [0.59; 3.64] 5.0%
Shaoyan Zheng 2021 15 195.17 7.7300 15 137.20 15.4600 4.61 [3.18; 6.05] 5.1%
Mahrous Abdelbasset Ibrahim 2021 8 12.88 0.4700 8 7.95 0.3500 11.25 [6.69; 15.80] 2.4%
Yasmen F. Mahran 2019 5 22.93 3.1600 5 11.54 2.1100 3.83 [1.37; 6.28] 4.1%
Yasmen F. Mahran 2019 5 23.09 4.7400 5 11.54 2.1100 2.84 [0.84; 4.84] 4.5%
Eman M. Mantawy 2018 10 266.12 42.9800 10 153.72 21.4900 3.17 [1.77; 4.57] 5.1%
Rauf Melekoglu 2018 10 62.20 6.2000 10 41.50 2.8600 4.11 [2.44; 5.77] 4.9%
Rauf Melekoglu 2018 10 67.20 3.0200 10 41.50 2.8600 8.37 [5.36; 11.37] 3.6%
Random effects model 98 98 3.26 [1.82; 4.70] 55.8%
Heterogeneity: I^2 = 84%, τ^2 = 5.4944, p < 0.01
subgroup = Mouse
Xianliang Luo 2023 8 54.27 5.7273 8 43.91 6.8182 1.56 [0.40; 2.71] 5.3%
Lingli Xin 2023 5 60.12 10.9034 5 34.58 6.8536 2.53 [0.66; 4.40] 4.7%
Mengwen Zhang 2022 5 10.82 1.0300 5 8.55 0.6700 2.36 [0.56; 4.16] 4.7%
Xinning Li 2022 20 38.06 3.1300 20 19.70 2.4600 6.39 [4.80; 7.99] 4.9%
Yuntao Zhao 2022 5 9.97 0.2900 5 7.57 0.9600 3.06 [0.96; 5.15] 4.4%
Ning Li 2019 10 11.26 2.0000 10 3.63 1.8300 3.81 [2.24; 5.39] 4.9%
Zhengjie Yan 2018 10 8.59 0.5400 10 5.65 0.3300 6.29 [3.96; 8.62] 4.2%
Yayun Wu 2018 14 55.61 12.6300 14 43.07 19.8400 0.73 [−0.04; 1.50] 5.5%
Lianli He 2017 25 41.46 6.4400 25 29.10 2.3200 2.51 [1.76; 3.27] 5.5%
Random effects model 102 102 3.13 [1.87; 4.40] 44.2%
Heterogeneity: I^2 = 86%, τ^2 = 3.0906, p < 0.01
Random effects model 200 200 3.16 [2.23; 4.08] 100.0%
Heterogeneity: I^2 = 84%, τ^2 = 3.8289, p < 0.01
Test for subgroup differences: χ^2_1 = 0.02, df = 1 (p = 0.90)
−15 −10 −5 0 5 10 15

H

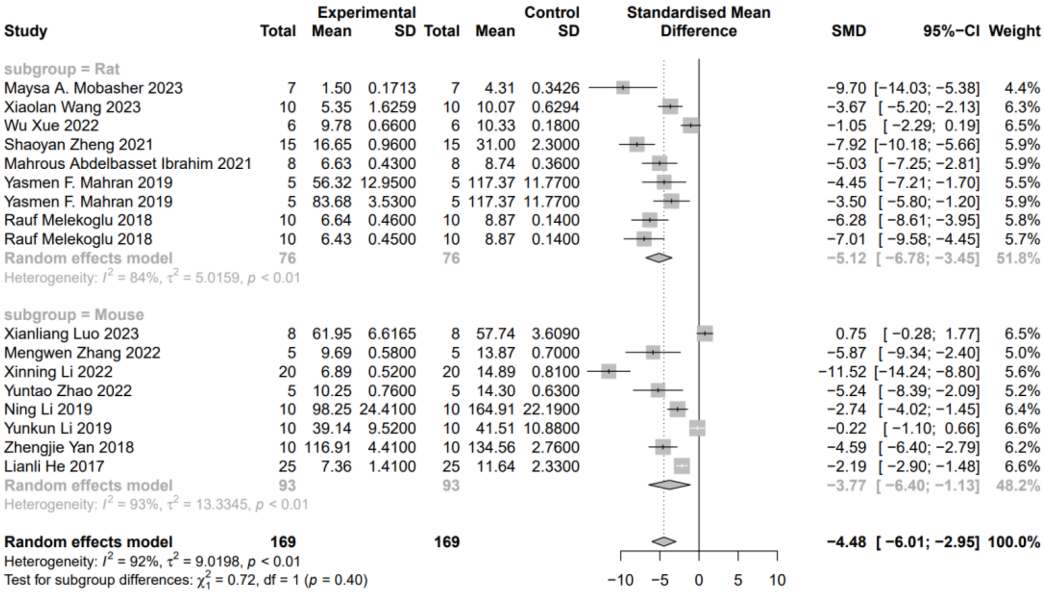

Study | Experimental Total | Mean | SD | Control Total | Mean | SD | Standardised Mean Difference | SMD | 95%-CI | Weight
subgroup = Rat
Maysa A. Mobasher 2023 7 1.50 0.1713 7 4.31 0.3426 −9.70 [−14.03; −5.38] 4.4%
Xiaolan Wang 2023 10 5.35 1.6259 10 10.07 0.6294 −3.67 [−5.20; −2.13] 6.3%
Wu Xue 2022 6 9.78 0.6600 6 10.33 0.1800 −1.05 [−2.29; 0.19] 6.5%
Shaoyan Zheng 2021 15 16.65 0.9600 15 31.00 2.3000 −7.92 [−10.18; −5.66] 5.9%
Mahrous Abdelbasset Ibrahim 2021 8 6.63 0.4300 8 8.74 0.3600 −5.03 [−7.25; −2.81] 5.9%
Yasmen F. Mahran 2019 5 56.32 12.9500 5 117.37 11.7700 −4.45 [−7.21; −1.70] 5.5%
Yasmen F. Mahran 2019 5 83.68 3.5300 5 117.37 11.7700 −3.50 [−5.80; −1.20] 5.9%
Rauf Melekoglu 2018 10 6.64 0.4600 10 8.87 0.1400 −6.28 [−8.61; −3.95] 5.8%
Rauf Melekoglu 2018 10 6.43 0.4500 10 8.87 0.1400 −7.01 [−9.58; −4.45] 5.7%
Random effects model 76 76 −5.12 [−6.78; −3.45] 51.8%
Heterogeneity: I^2 = 84%, τ^2 = 5.0159, p < 0.01
subgroup = Mouse
Xianliang Luo 2023 8 61.95 6.6165 8 57.74 3.6090 0.75 [−0.28; 1.77] 6.5%
Mengwen Zhang 2022 5 9.69 0.5800 5 13.87 0.7000 −5.87 [−9.34; −2.40] 5.0%
Xinning Li 2022 20 6.89 0.5200 20 14.89 0.8100 −11.52 [−14.24; −8.80] 5.6%
Yuntao Zhao 2022 5 10.25 0.7600 5 14.30 0.6300 −5.24 [−8.39; −2.09] 5.2%
Ning Li 2019 10 98.25 24.4100 10 164.91 22.1900 −2.74 [−4.02; −1.45] 6.4%
Yunkun Li 2019 10 39.14 9.5200 10 41.51 10.8800 −0.22 [−1.10; 0.66] 6.6%
Zhengjie Yan 2018 10 116.91 4.4100 10 134.56 2.7600 −4.59 [−6.40; −2.79] 6.2%
Lianli He 2017 25 7.36 1.4100 25 11.64 2.3300 −2.19 [−2.90; −1.48] 6.6%
Random effects model 93 93 −3.77 [−6.40; −1.13] 48.2%
Heterogeneity: I^2 = 93%, τ^2 = 13.3345, p < 0.01
Random effects model 169 169 −4.48 [−6.01; −2.95] 100.0%
Heterogeneity: I^2 = 92%, τ^2 = 9.0198, p < 0.01
Test for subgroup differences: χ^2_1 = 0.72, df = 1 (p = 0.40)
−10 −5 0 5 10

I

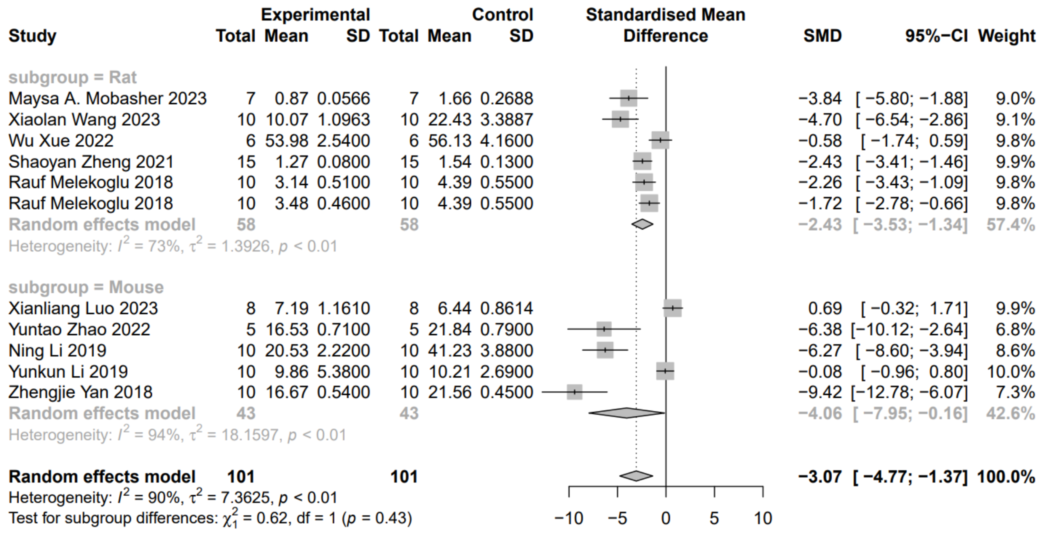

J

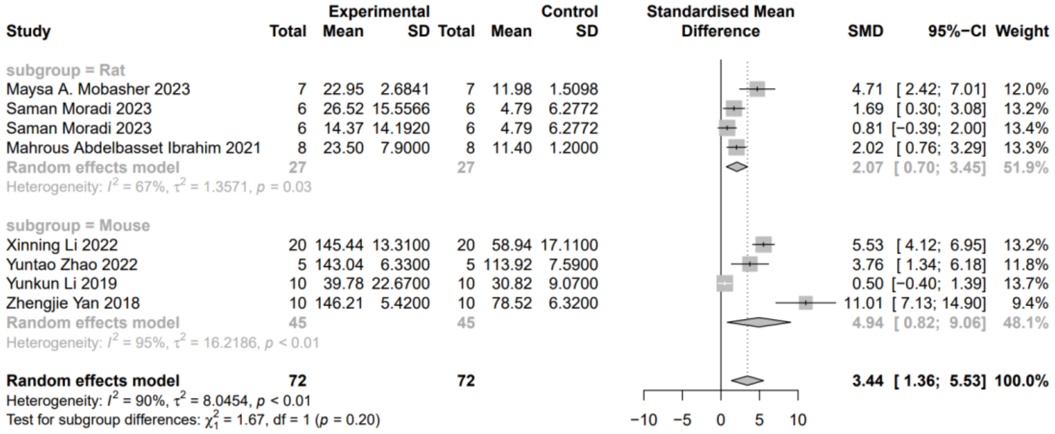

Figure S2.3. Forest plots: subgroup analysis of the strains. (A) counts of primordial follicles, (B) counts of primary follicles, (C) counts of secondary follicles, (D) counts of antral follicles, (E) counts of atretic follicles, (F) AMH, (G) E2, (H) FSH, (I) LH, (J) P.
